# Uncovering system-specific stress signatures in primate teeth with multimodal imaging

**DOI:** 10.1038/srep18802

**Published:** 2016-01-04

**Authors:** Christine Austin, Tanya M. Smith, Ramin M. Z. Farahani, Katie Hinde, Elizabeth A. Carter, Joonsup Lee, Peter A. Lay, Brendan J. Kennedy, Babak Sarrafpour, Rosalind J. Wright, Robert O. Wright, Manish Arora

**Affiliations:** 1 Senator Frank R. Lautenberg Environmental Health Sciences Laboratory, Department of Preventive Medicine, Icahn School of Medicine at Mount Sinai, New York, New York 10029, USA; 2 Faculty of Dentistry, The University of Sydney, Sydney, New South Wales 2006, Australia; 3 Department of Human Evolutionary Biology, Harvard University, Cambridge, Massachusetts 02138, USA; 4 School of Human Evolution and Social Change, Center for Evolution and Medicine, Arizona State University, Arizona, 85287, USA; 5 California National Primate Research Center, Davis, California 95616, USA; 6 Vibrational Spectroscopy Core Facility, The University of Sydney, Sydney, New South Wales 2006, Australia; 7 School of Chemistry, The University of Sydney, Sydney, New South Wales 2006, Australia; 8 Department of Pediatrics, Icahn School of Medicine at Mount Sinai, New York, New York 10029, USA

## Abstract

Early life stress can disrupt development and negatively impact long-term health trajectories. Reconstructing histories of early life exposure to external stressors is hampered by the absence of retrospective time-specific biomarkers. Defects in tooth enamel have been used to reconstruct stress but the methods used are subjective and do not identify the specific biological systems impacted by external stressors. Here we show that external physical and social stressors impart biochemical signatures in primate teeth that can be retrieved to objectively reconstruct the timing of early life developmental disruptions. Using teeth from captive macaques, we uncovered elemental imprints specific to disruptions of skeletal growth, including major disruptions in body weight trajectory and moderate to severe illnesses. Discrete increases in heat shock protein-70 expression in dentine coincided with elemental signatures, confirming that elemental signals were associated with activation of stress-related pathways. To overcome limitations of conventional light-microscopic analysis, we used high resolution Raman microspectral imaging to identify structural and compositional alterations in enamel and dentine that coincided with elemental signatures and with detailed medical and behavioural data. Integrating these objective biochemical markers with temporal mapping of teeth enables the retrospective study of early life developmental disruptions and their ensuing health sequelae.

External stressors that are real or perceived can disrupt physiologic pathways and elicit mechanisms that attempt to restore homeostasis^1^,^2^,^3^. Exposure to such adverse stimuli during foetal and early childhood periods may alter life-long health trajectories, increasing the risk of neurodevelopmental disorders, cardiovascular disease and cancer^4^. However the absence of retrospective biomarkers of stress events during early life is a major challenge for identifying critical developmental windows of heightened susceptibility to external stressors.

The prenatal and early childhood periods represents a critical window during which insult from environmental stressors can have lifelong consequences^5^,^6^,^7^,^8^. The ability to adapt to environmental cues during development is an important factor in the success of an individual and species. However, a mismatch between the early life environment adapted to and the adult environment actually experienced can lead to adverse outcomes^5^,^9^,^10^. In addition, stress response systems undergo phased development from early prenatal life to adolescence, and repeated exposure to stress during these periods can disrupt development and ultimately dysregulate and compromise the resilience of these systems, producing poorly controlled responses that are over-reactive or slow to down-regulate once activated^11^,^12^. Dysregulated stress responses can have far-reaching consequences including suppression of the immune system, leaving individuals vulnerable to infections and other chronic health disorders^13^,^14^, and increased susceptibility to neurodevelopmental deficits from environmental chemicals^15^.

Exposure timing is important as it determines which organs or systems are affected and to what degree^5^,^16^. The brain is particularly susceptible to insult as development begins prenatally and extends over two decades, a long period of vulnerability during which different systems or regions of the brain are vulnerable at different time points^17^. Prolonged exposure to stress hormones can impact neurodevelopment and impair brain function, and the timing of early life stress determines the functions impacted^7^,^18^. Therefore, studies of the health effects of stress need not only consider the intensity of stress events, but also the timing of such events.

Teeth are a promising system to investigate the history of early life developmental disruptions because the hard tissues (enamel and dentine) contain a daily record of their development. External stressors may disrupt enamel and dentine matrix deposition, resulting in permanent accentuated lines in fully mineralized teeth (akin to prominent growth rings), and the precise chronological ages of these features may be determined after registry with the neonatal (birth) line^19^,^20^,^21^,^22^. Past studies have used this property to compare the timing of clinical events such as injuries, bouts of dehydration/diarrhoea, and hospitalisations, to accentuated lines^20^,^21^,^22^. However, the response to adverse stimuli involves complex neurobiological mechanisms, and current approaches do not identify the biological systems or pathways impacted by external stressors. Furthermore, traditional methods that rely solely on light microscopy to examine accentuated lines are subjective, and highly dependent on operator expertise, quality of sample preparation and microscopy technique^22^,^23^,^24^. These methods have primarily been applied to enamel, as identifying accentuated lines in dentine is more challenging^23^,^24^.

Here we employ a novel multi-tiered approach to document the efficacy of primate teeth for accurate retrospective records of stress. First, we provide objective elemental analysis to determine the presence of disruptions specific to the skeletal system. Second, we identify molecular markers of specific homeostatic pathways activated in response to external stressors, which can be retrieved from teeth to determine the presence of past developmental disruptions. Finally, we demonstrate a high-resolution chemical imaging methodology that is objective and quantifiable, requires limited sample preparation, provides fine temporal detail and is effective in analysing both enamel and dentine. We also compare traditional light microscopic analysis used to precisely age accentuated lines to our chemical biomarkers.

## Results

### Elemental imprints of skeletal growth trajectory disruptions

**Figure 1:**
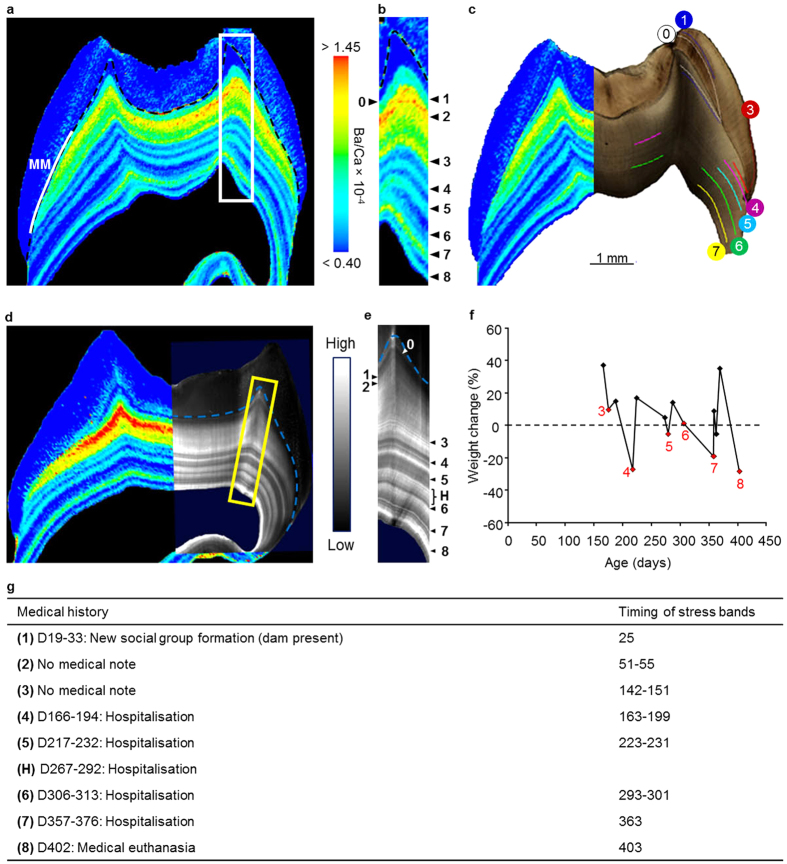
Biochemical signatures in MMU401 permanent first molar during multiple stress events. (**a**) Ba distribution map (as Ba/Ca) shows multiple bands of increased concentration. Nursing signal is also evident (MM)^42^. Timing of Ba bands was determined by histological analysis in (**c**). (**b**) Area highlighted in (**a**). Discrete bands of high Ba are shown by arrowheads numbered 1 to 8 corresponding to this animal’s medical history (**f**,**g**). (**c**) Light micrograph with accentuated lines labelled according to **g**. The neonatal line is labelled 0. (**d**) Grey scale Raman microspectroscopic map showing variation in the organic biomolecular matrix (874 cm^−1^ band intensity). Raman analysis was performed on the opposing face tooth block of the thin section in (**a**–**c**). Elemental analysis was also performed on this block and the Ba/Ca map has been overlaid to show correlation of chemical bands. (**e**) Area highlighted in (**d**). Discrete bands are shown by arrowheads corresponding to events in medical history. Additional bands not observed in the light microscopy map are visible, for example band H. (**f**) Percent weight change over consecutive measurements as proxy for skeletal growth trajectory. Severe disruptions in normal weight gain trajectory are indicated by numbers coincident with medical events (**g**), bands of increased Ba (**b**), accentuated lines apparent under light microscopy (**c**), and accentuated lines in the Raman map (**e**). Weight measurements were not available before 150 days of age. (**g**) Summary of animal’s medical history. Event numbers correspond to data in (**b**,**c**,**e**).

**Figure 2:**
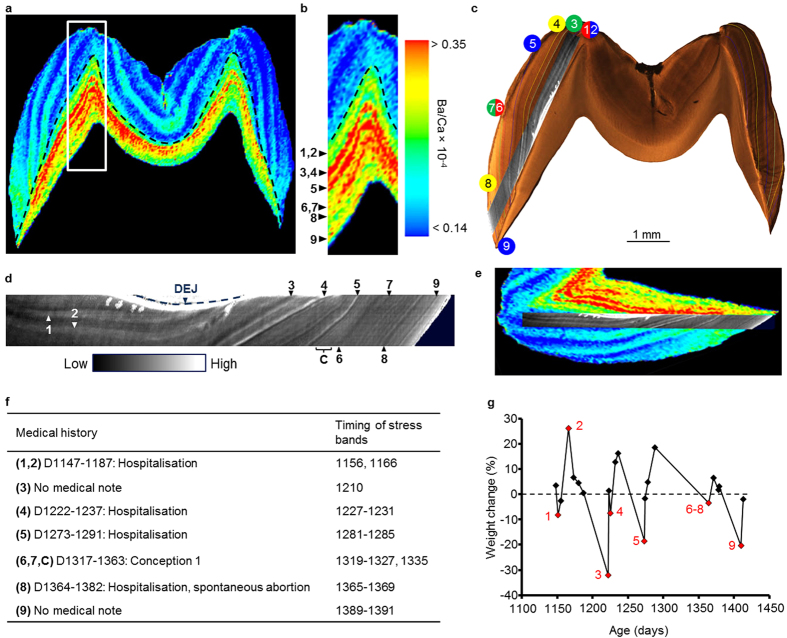
Biochemical signatures in MMU336 permanent third molar during multiple stress events. (**a**) Ba distribution map (as Ba/Ca) shows multiple bands of increased concentration. Timing of Ba bands was determined by histological analysis in (**c**). (**b**) Area highlighted in (**a**). Discrete bands of high Ba are shown by arrowheads numbered 1 to 9 corresponding to this animal’s medical history (**f**,**g**). (**c**) Light micrograph of third molar showing accentuated lines (developmental timing of these lines is shown in **f**). The section of enamel analysed by Raman microspectroscopy is overlaid and shows close agreement with accentuated lines in enamel. (**d**) Enlarged section of Raman microspectroscopic map shown in (**c**) with discrete bands shown by arrowheads corresponding to events in medical history (**f**). (**e**) Overlay of Ba/Ca map from **a** and Raman map from **d** showing correlation between the two techniques. Raman maps shows additional bands due to finer resolution. (**f**) Summary of macaque’s medical history. Event numbers correspond to data in panels (**b−d**). (**g**) Percent weight change over consecutive measurements as proxy for skeletal growth trajectory. Severe disruptions in normal weight gain trajectory are indicated by numbers coincident with medical events (**f**), bands of increased Ba concentration (**b**), accentuated lines apparent under light microscopy (**c**), and accentuated lines in the Raman map (**d**).

Stress events such as illness or nutritional disruptions can precipitate reliance on somatic stores^25^, which may be difficult to detect months or years later. Probe-free elemental and biochemical imaging can provide novel information on the molecular basis of disease processes and changes in physiology^26^. We found biomarkers corresponding to growth trajectory disruptions within teeth of eight captive juvenile macaques (*Macaca mulatta*) using laser ablation-inductively coupled plasma-mass spectrometry for elemental analysis. Bands of increased barium (Ba normalized to calcium, Ba/Ca) in dentine corresponded to major disruptions in body weight trajectory (relative percent weight change over consecutive measurements), illnesses noted in the animal’s medical history, and accentuated lines in enamel and dentine observed with light microscopy (Figs 1 and 2). Seventy percent of all hospitalisations (17 of 24) and 67% of biobehavioural assessments (4 of 6) corresponded with increased Ba/Ca or Pb/Ca bands. No band was observed around the time of the biobehavioural control separation for MMU152. Bands were also observed in two individuals (MMU515 and MMU151) around the time their mothers conceived subsequent offspring while they were in close association (Supplementary Table S1 and Supplementary Fig. S1), and around the time a young female macaque (MMU336) conceived and at the time of spontaneous abortion (see Fig. 2).

Of 17 major, negative disruptions to weight gain trajectory recorded during the respective period of tooth development, 16 corresponded to bands of increased Ba/Ca during the respective periods of tooth development (94%; see methods section for details). In 15 instances, bands of increased Ba/Ca correlated with a weight gain (of 34 recorded weight gains), representing additional evidence that Ba signatures are linked to major transitions in skeletal growth that are beyond homeostatic norms. Additional bands were observed in some samples where there were no medical notes (Supplementary Table S1). This was expected as any subclinical skeletal disruptions without visible symptoms would not have been noted in the medical history^20^,^22^.

In all samples, elemental banding was narrower and better differentiated in dentine than enamel. This is due to the greater lag between matrix secretion and mineralization in enamel when compared to dentine^27^. Strontium (Sr/Ca) levels in dentine also showed close correspondence to medical histories (Supplementary Fig. S2, S3). Co-localization of discrete increases in dentine Ba/Ca (and Sr/Ca) with increases in lead (Pb/Ca) concentration strongly suggests that these elements were released from skeletal stores (Supplementary Fig. S2). Over 95% of absorbed Pb is stored in the skeletal compartment^28^, and the macaques had no noted exposure to Pb from diet or medication when body weight and/or illness were recorded. Discrete banding was also observed in Zn/Ca distribution maps, with some bands correlating with increased Ba/Ca bands (Supplementary Fig. S2 and S3). About 60% of the body’s Zn is stored in muscles and only 30% in bone^29^, therefore Zn levels are likely less sensitive to disruptions of skeletal growth trajectories than Ba or Sr, which are primarily stored in bones. Overall, Ba generally showed the most reliable banding patterns in relation to medical histories but the measurement of multiple elements provided complimentary data with some bands more clearly observed using Sr, Pb or Zn.

### Stress-related signatures in dentine

**Figure 3:**
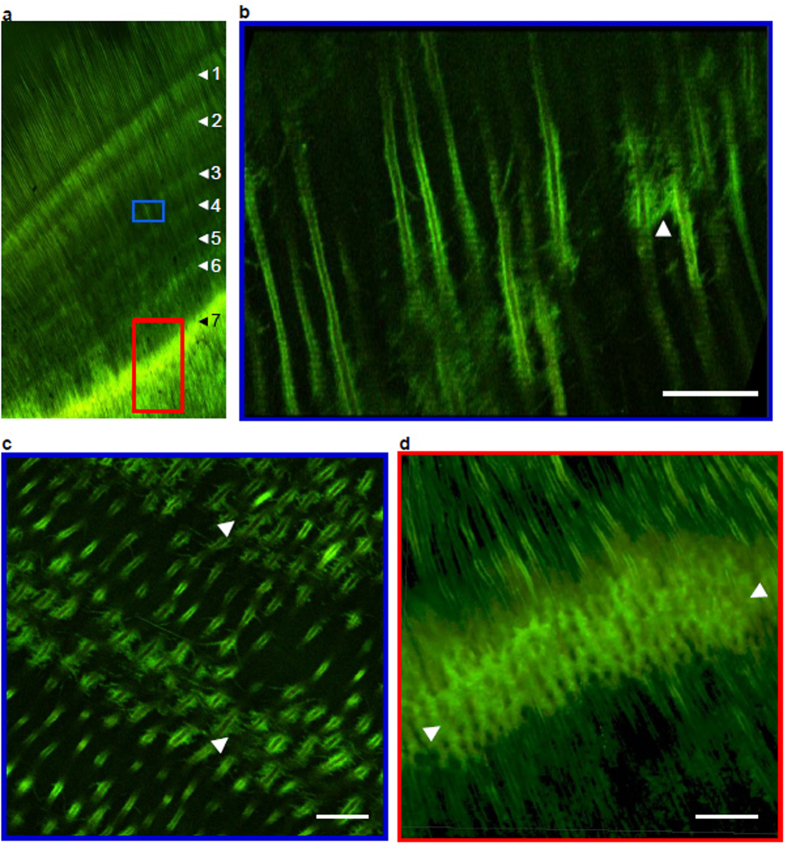
Imprint of stress events in dentine confirmed by heat shock protein (HSP70) expression. (**a**) HSP70 distribution in dentine of MMU401 molar shown in Fig. 1. Seven increases in HSP70 content coincide with events shown in Fig. 1b,e. Variations in intensity of HSP expression are apparent; low expression, blue rectangle, and high expression, red rectangle. (**b**) High-resolution micrograph of dentine tubules in area highlighted in blue box in **a.** Structure of dentinal tubules shows minor disruptions with appearance of lateral strands (one such area is indicated by arrowhead). (**c**) Oblique view of area showing moderate expression of HSP70. Lateral strands can be seen in areas of stress lines (arrowheads). (**d**) High-resolution micrograph of dentine tubules in area highlighted in red in **a.** Complete disruption of tubular structure in an area of high HSP70 content corresponding to a stress event (bounded by arrowheads), with normal tubular structure before and after the event. Scale bars = 20 μm.

Of the many complex neurobiological pathways involved in stress responses, we targeted the 70 kDa heat shock protein (HSP70). Cultured odontoblast-like cells^30^ and dental pulp cells^31^ express HSP70 under stress, and HSP70 are particularly sensitive to external stressors and environmental chemicals^32^,^33^. HSP70 and other heat shock protein families have also been directly detected in human dentine^34^,^35^. Heat shock proteins are molecular chaperones that are released to cope with the stress-induced denaturation of other proteins^36^ and are classified into several subfamilies based on molecular weight^37^,^38^. The HSP70 family of proteins are present at low basal levels, and increase in response to environmental and physiological stressors such as elevated temperatures, oxidative stress and heavy metal exposure^38^,^39^. We found that HSP70 was deposited on the periphery of dentinal tubules, presumably in the peritubular dentine (Fig. 3c and Supplementary Fig. S4). Figure 3a shows seven distinct bands of higher HSP70 content in the dentine of the molar in Fig. 1. These increases in HSP70 content corresponded to 7 of 8 elemental signatures related to accentuated lines and medical data (Fig. 1). Furthermore, confocal microscopy revealed a mild disruption of dentine tubular structure with horizontal processes in bands of moderately increased HSP70 content (Fig. 3b,c). In regions where HSP70 expression was highest, the columnar pattern of dentine tubules was completely disrupted (Fig. 3d). Lower magnification images of HSP70 expression are included in Supplementary Fig. S5.

### High-resolution Raman spectral imaging of stress signatures in teeth

Alterations in enamel and dentine matrix secretion during stress events are likely to result in structural and compositional alterations in mineralized teeth that serve as objective signatures of the timing of past developmental disturbances. High-resolution Raman spectral imaging revealed fine scale biomineral and molecular imprints that correspond to variations in elemental concentrations, events recorded in the animals’ medical histories and accentuated lines observed with light microscopy (Figs 1 and 2). Discrete banding was observed as minor changes in the biomineral structure, organic biomolecular content and protein conformations of dentine relative to adjacent healthy dentine (Supplementary Fig. S6, S7, Supplementary Table S1 and Supplementary Discussion). These differences were confirmed with a principle component analysis (Supplementary Figs S8–S10). Of the 21 hospitalisations that occurred during the study period, 17 corresponded to discrete bands in Raman maps (81%). Of 23 weight losses occurring during the period covered by Raman maps, 19 corresponded with a Raman band (83%). Only 1 of 3 biobehavioural assessments included in the Raman analysis corresponded to Raman bands (see Methods section). Raman bands were observed during the period when mothers of MMU515 and MMU151 were pregnant with subsequent offspring (Supplementary Table S1 and Supplementary Fig. S1), and during the time MMU336 conceived, was pregnant and spontaneously aborted (Fig. 2 event C, Supplementary Table S1).

As with our elemental analysis, additional bands were observed in some individuals that did not correspond to events in medical records. Importantly, Raman microspectroscopic analysis revealed fine-scale features in dentine that were not visible using traditional light microscopy (Supplementary Fig. S11). These features appear to represent a combination of regular, rhythmic incremental lines as well as aperiodic accentuated lines. Additional data for all nine animals showing correlation between different biochemical signals and medical records is included in the Supplementary Information.

### Traditional light microscopy

Accentuated lines aged using light microscopy showed broad agreement with medical records (Figs 1 and 2, Supplementary Table S1). Sixty-six percent of hospitalisations (16 of 24) corresponded with accentuated lines formed during the period covered by histological maps. Light microscopy revealed that four of six individuals who underwent biobehavioral assessments showed corresponding accentuated lines (typically within a day of assessment), and the remaining two individuals (MMU325 and MMU151) also appeared to show corresponding accentuated lines, although the associations were not consistent across multiple cusps. MMU152, the control animal for biobehavioural assessments did not consistently show an accentuated line under light microscopy around the time of the event. Accentuated lines were observed during the period mothers of MMU515 and MMU151 were pregnant with subsequent offspring (Supplementary Table S1 and Supplementary Fig. S1) and during the period MMU336 conceived, was pregnant and aborted (Fig. 2 and Supplementary Table S1). In some instances accentuated lines were identified that did not correspond to colony records, suggesting that some minor experiences or subclinical states, as yet unexplained, disrupt tooth development.

## Discussion

**Figure 4:**
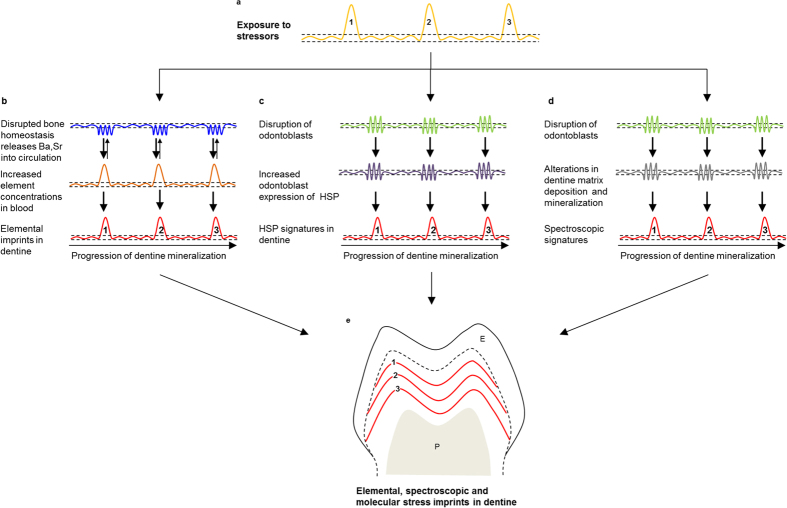
Conceptual framework of biochemical imprints in dentine. (**a**) Three discrete hypothetical exposures to external stressors (labelled 1–3) which cause bodily systems to depart from homeostasis (dashed lines). (**b**) Disruption of skeletal homeostasis in response to stress events. Bone and blood elemental levels are in equilibrium under homeostasis. When external stressors disrupt bone remodelling, a net increase in elemental transfer to blood results in increased elemental deposition at the mineralizing front of dentine as discrete bands associated with the timing of stress events (**e**). (**c**) Disruption of odontoblastic homeostasis results in increased HSP70 content in dentine matrix. Higher levels of HSP70 during stress events results in a banding pattern evident in dentine. (**d**) Disruption of odontoblast function in response to external stressors will result in the deposition of an altered biomineral matrix composition. Changes in carbonate substitution or protein conformation will imprint a molecular signature in discrete regions of dentine formed during the stress exposure. (**e**) Representation of biochemical imprints in dentine that can be directly linked to stress events 1–3 shown in (**a**). Enamel (E) and pulp (P) are not depicted.

The archival nature of enamel and dentine, which captures and preserves important aspects of developmental history, provides an opportunity to retrospectively study health outcomes in response to early life stress. We have shown that external physical and social stressors such as injury, infections and separation from dam can impart permanent biochemical signatures in primate enamel and dentine (Fig. 4). Traditional light microscopy-based methods of visualizing accentuated lines in enamel are more subjective than elemental approaches, and are non-specific to the systems affected by external stressors. Moreover, accentuated lines do not provide insight into the molecular pathways activated during a stress event, which limits their application in studies of developmental stress and the origins of health disorders during early life.

We identified imprints sensitive to the disruption of skeletal homeostasis using spatiotemporal analysis of trace elements in dentine, representing a novel approach to study growth trajectories. Ba, Sr, and Pb are predominantly stored in the skeletal system, and during homeostasis skeletal stores of these metals reach equilibrium with blood concentrations^40^,^41^ (Fig. 4b). When external stressors disrupt normal bone remodelling and cause acute mineral loss, the increased release of skeletal Ba and Sr (and Pb in exposed individuals) elevate blood concentrations, which are imprinted at the tooth mineralization front as bands of high element concentrations (Fig. 4b,e). These acute elemental bands are unlikely to arise from diet or environmental causes. The animals studied were housed in a controlled environment where Ca, Ba, or Sr intake from diet or environmental exposure did not vary dramatically over a short time-period or across subjects. Furthermore, Pb is a well-established toxicant and is not present in pharmaceuticals in amounts that would cause sudden increases during hospitalisations. We have previously shown that elemental signatures from diet, such as transitions from mother’s milk to other foods, are very different from the acute banding pattern studied here^42^.

Of the elements analysed (Ba, Sr, Pb and Zn), Ba generally showed the most reliable banding patterns in relation to the medical histories. Ba, Sr and Pb followed similar banding patterns with some differences in relative intensity between bands. These differences may be due to different baseline levels across the tooth or different transport mechanisms between metals. These differences have also been observed in studies of diagenesis that found Sr was generally more susceptible to exchange between bone and soil than Ba^43^. Overall, Sr banding tended to be clearer in later forming dentine (post-weaning) while Pb banding was less clear in animals with low exposure. Due to the different distribution of Zn in the body compared to Ba, Sr and Pb, we propose that Zn banding may point to other pathways. Although we found Ba to be the most reliable stress signal, we encourage multi-elemental analysis for the best identification of stress signals.

During stress events, odontoblasts express stress mediators such as HSP70 in the dentine matrix that become archived in the mineralized tissue (Fig. 4c). Co-localization of increased HSP70 expression with elemental signatures confirms that specific homeostatic pathways are activated during developmental disruptions. This is supported by the absence of increased HSP70 at the neonatal line, a prominent stress line in enamel and dentine formed at birth that is unlikely to coincide with skeletal growth disruption. HSP70 expression is known to increase in response to heavy metal exposure and therefore we cannot rule out that the increased HSP70 expression was in response to the elevated endogenous metals (Ba, Sr, Pb) released during bone remodelling. However, the absence of an increase in HSP70 across the neonatal line, where these metals also increase, indicates that HSP70 levels in dentine may increase in response to other stressors as well. Therefore, there is potential to target the disruption of specific homeostatic pathways based on the protein selected^38^. Details of the biomolecular pathways relevant in HSP70 dentine deposition and alteration of dentine structure observed at the stress lines are given in the Supplementary Discussion. Our analysis of dentine HSP70 profiles provides a framework that may be applied to study other molecular targets of stress-related pathways that may be found in teeth.

Disruption of odontoblastic function caused by stress events results in alterations in dentine matrix deposition and mineralization (Fig. 4d)^44^, which imparts biochemical signals in mineralized dentine. We demonstrate that these signals can be observed as modification of the biomineral apatite (carbonate substitution) or organic biomolecular matrix (protein content or conformation) (Fig. 4d). Using Raman microspectroscopy we capture temporal variations in the molecular composition of enamel and dentine that correspond to elemental and temporal maps, and the animals’ history of illness. The higher resolution of Raman microspectroscopy, compared to LA-ICP-MS analysis, has the advantage of measuring the width and thus the precise duration of stress events. Combined with signal intensity this technique has the potential to measure the intensity and duration of stress events objectively. The finer level of detail evident in the objective quantitative nature of Raman microspectroscopic analysis of enamel and dentine provide distinct advantages over conventional light microscopy.

All of our analytical approaches revealed some accentuated lines/bands for which there was no corresponding medical data. This is expected because medical records include only events that cause clinically evident symptoms for which treatment is administered, not each physical or social stressor. Previous studies have also observed accentuated lines using light microscopy that did not correspond to recorded medical events^20^,^22^,^45^, ecological variation^45^, or community-level disease outbreaks^45^. Multi-modal imaging of teeth may, therefore, provide a more complete record of an individual’s stress history than medical or behavioural records alone. Furthermore, we observe differences in stress signatures depending upon the imaging technique. For example, the Raman spectroscopic maps revealed many more accentuated bands than immunohistochemical mapping of HSP70 or elemental profiles. This is expected because the methods we employ target different stress-related pathways and physiologic responses to stress.

The techniques described here could also be used to study indicators of stress in bone, another tissue that mineralises incrementally. Researchers have observed stress-induced growth disruptions in skeletal remains, however multiple concerns with their identification and interpretation have been raised^46^,^47^,^48^. The analytical techniques presented here could yield similar results in bone and identify signatures of specific stress pathways. However, unlike teeth, bone is actively remodelled and therefore the absorption of stress lines formed throughout life is possible^49^.

There is immense variability in the stress-related response among individuals^1^,^50^, which is not captured by questionnaires. The chemical and molecular signatures that we present here from a small biomedical animal model are an objective measure of the stress response, and when applied to larger studies will help unravel the health outcomes associated with stress exposure. A major advantage of chemical analysis of accentuated lines in teeth is the quantitative nature of these techniques that can be used to quantify the intensity of stress responses in future studies. Importantly, these methods could measure individuals’ level of responsivity to stress across a population experiencing the same challenges. For example, not all of the animals studied here demonstrated the same signatures for similar events such as relocation, hospitalisation, biobehavioural assessment or first physical exam. This may indicate different animals responded to the same stress events to different degrees. Measurement of an individuals’ response to stress would greatly enhance studies on the health effects of stress.

Biological organisms must allocate resources to the competing biological imperatives of maintenance, development and reproduction^14^. Such allocation is a function of life stage and environmental factors, and trade-offs are inevitable as resources used for one purpose cannot be used for another. These trade-offs underlie the signatures of early life programming; response to an environmental cue or stressor is the reallocation of resources to different somatic priorities which, while necessary for adaptation and survival, can handicap some physiological functions later in life^10^,^51^. Interest in elucidating the environmental cues or external stressors that induce adaptation is growing, and stress, illness, under-nutrition and chemical exposures have been linked to adverse consequences of adaptation^5^,^8^,^14^,^52^. Studies are hindered by the lack of a biomarker capable of reconstructing the stress history of individuals. Biochemical signals in teeth have the potential to not only reconstruct the stress response history of an individual from the second trimester to early childhood in the case of deciduous teeth, and into adulthood for permanent teeth, but also to reconstruct exposure to metals, organic chemicals^53^ and diet^42^. Teeth can, therefore, provide a more comprehensive description of early life for use in epidemiologic and public health studies, as well as studies of primate evolution and bioarchaeology that seek to document the health of extinct populations^54^.

The integration of objective biochemical markers detailed here provides an important tool for unravelling the complex interactions between exposure to external stressors, disruption of physiologic pathways, and activation of mechanisms to restore homeostasis, ultimately leading to a better understanding of the role of early life stress in long-term health trajectories.

## Materials and Methods

### Samples

First and third molars were obtained opportunistically during necropsies of juvenile macaques at the California National Primate Research Center (*Macaca mulatta*)^42^. Of the nine subjects, N =8 were born in the outdoor breeding colony in large enclosures (0.2 hectares) in complex social groups composed of close kin, distant kin, and non-kin. Visual inspections for clinical symptoms of each animal were performed each morning of the animal’s life and referred to Primate Medicine for injury or illness. On average, every 4 months each animal was sedated and received a physical exam. Typically during their first physical exam, their identification number is tattooed on their leg for accurate record-keeping. Treatment of animals was carried out in accordance with the approved guidelines of the California National Primate Research Center by Primate Medicine personnel. Experimental protocols were approved by the Institutional Animal Care and Use Committee at University of California Davis. This study made use of existing archival records and did not do any experimental research on living animals.

Histological sections were prepared according to standard protocols^55^, and accentuated lines in the coronal enamel and dentine were spatiotemporally mapped (Supplementary Table S1). Developmental defect occurrences were converted to specific chronological ages by determining postnatal developmental time, which was assessed from counts and measurements of daily cross-striations along a prism track from the neonatal (birth) line (labelled as 0 on histological images) to the accentuated line^23^. Ages were assigned to accentuated lines in the lateral enamel by adding time represented by preceding long-period growth lines to the age at cusp formation. Not all accentuated lines were observed across all sections from the same animal. Ages are accurate to within a few days to weeks of actual events depending on the quality of the section and clarity of incremental features^23^,^42^. For MMU336, ages of accentuated lines were determined by working backwards from the cessation of tooth formation at death, as third molar formation begins after birth and therefore the neonatal line was not available.

Histological and chemical analyses were performed in a blinded manner independent of the medical histories. Our primary aim when comparing aged accentuated lines to medical histories was to assess the concurrence of accentuated lines and major events such as hospitalisations and biobehavioural assessments. Illness or other events without clinical symptoms would not have been recorded in the animals’ medical histories. Animal relocations, morphometrics (mass), veterinary treatments, and biobehavioural assessments were recorded, and events matched to independently-aged accentuated lines. MMU401 did not undergo biobehavioural assessment, nor did MMU336 during the period of third molar formation. Biobehavioural assessments are a standardised 25-hr period of separation from the mother and social group, where individuals are relocated to a novel setting and experience a series of behavioural and physiological assessments described in detail elsewhere^56^. MMU152 was relocated for biobehavioural assessment but did not undergo assessment to act as a control.

Weight change plots in Figs 1 and 2 and Supplementary Fig. S1 include all documented weights during the period of tooth formation for each animal. Weight changes closest to histologically-aged accentuated lines are marked in red with the corresponding label. Weighings varied in number and frequency among animals. When comparing weight loss with elemental and Raman bands, we excluded MMU473 and MMU542 due to too few data points. For elemental bands, weight records <20 days apart were combined as this was considered below the resolution of elemental mapping.

### Elemental analysis

We used a New Wave Research NWR-193 laser ablation system connected to an Agilent Technologies 7700s ICP-MS by Tygon® tubing. Details of our analytical methods have been published previously^42^. In brief, a 35 μm diameter laser beam was rastered along the sample surface in a straight line at a speed of 70 μm s^−1^, producing data points that correspond to a pixel size of approximately 35 × 35 μm. Element ratios (Ba/Ca, Sr/Ca, Pb/Ca and Zn/Ca) were calculated from concentrations determined using NIST 1486 bone meal as a standard and ^43^Ca, ^66^Zn, ^88^Sr, ^138^Ba and ^208^Pb isotopes. For elements not certified in NIST 1486, an average concentration calculated from determinations in two other studies^57^,^58^ was used. Elemental maps were processed using Interactive Spectral Imaging Data Analysis Software (ISIDAS), a custom-built software tool written using Python programming language, and MayaVi2 (Enthought Inc.), an open source data visualization application. Colour scales were applied using the linear blue–red LUT. Image backgrounds were converted to black (absent from the colour intensity scale) to clarify sample boundaries from the substrate. Elemental maps were overlaid on light microscopy images using the Georeferencer tool in QGIS (version 2.8.2-Wien). Reference points were placed along the outer edge and along the dentine-enamel junction of the tooth and the Thin Plate Spline transformation applied.

### Raman Microspectroscopic analysis

Embedded tooth blocks adjacent to histological thin sections (MMU401, MMU515, MMU152) or thin sections (MMU336, MMU325, MMU151) were analysed using a Renishaw Raman InVia Reflex Microscope (Renishaw plc., Wotton-under-Edge, UK), equipped with an air-cooled charge-coupled device (CCD). The spectrometer is fitted with holographic notch filters and two gratings (1200 mm/line (visible) 2400 mm/line (NIR)). The attached microscope is a Leica DMLM equipped with four objectives (×100/0.75 NA, ×50/0.75 NA, ×20/0.40 NA, ×5/0.12 NA) and a trinocular viewer that accommodates a video camera.

Sample excitation was achieved using a NIR laser (Renishaw plc., Wotton-under-Edge, UK) emitting at 785 nm. The spectrometer was controlled by PC with instrument control software (Renishaw WiRE™, Version 4.0).

Raman maps were collected in StreamLine**™** mode over the spectral range of 1810–724 cm^−1^ with a laser power of 15 mW (point mode) and using the following parameters; ×50 objective, 5.7 μm step size, exposure time of 2.3 s and Y binning of 4. The high resolution map of MMU401 was produced using the ×100 objective, a step size of 1.4 μm and Y binning of 2. Cosmic rays were removed and noise filtering applied using the WiRE v4.0 software. Curve fitting was then applied before generating images of the area, intensity, width and position of different bands. Principle component analysis was performed using The Unscrambler^®^ X (v10.3, CAMO Software) and WiRE 4.0 was used to generate principle component images.

When analysing dentine with Raman microscopy, we used the facing tooth block (since Raman spectroscopic signatures in dentine were obscured when using thin sections mounted on glass slides). Consequently, some Raman bands could not be aged with the same precision as those accentuated lines viewed under light microscopy of thin sections. However, we were able to assign temporal information to Raman bands by overlaying them with elemental bands that were visible in both the thin tooth sections and the facing tooth blocks. Raman analysis was not performed on MMU619, MMU542 or MMU473 due to the obliqueness of facing tooth blocks.

### Immunohistochemistry analysis

We conducted a standard immunohistochemical staining procedure, modified for novel application to dentine, to confirm that the elemental signatures coincided with activation of HSP-related homeostatic pathways. Histological tooth sections were treated in EDTA solution (10%, pH 8.0) as an antigen retrieval/light-demineralization step. Sections were then incubated in a blocking buffer of normal goat serum and Triton X-100 (10%) in phosphate buffered saline (0.01 M, PBS). A 10 μg mL^−1^ solution of monoclonal mouse anti-HSP 70 antibody (5A5) raised against its N-terminal portion (Abcam) was prepared using a modified blocking buffer as diluent (1% normal goat serum and 3% Triton X-100 in PBS). Samples were incubated with primary antibody overnight at 4 °C and then washed three times in PBS solution (0.01 M). Secondary goat anti-mouse antibody (conjugated to Alexa-488) was applied at 2 μg mL^−1^. Sections were incubated with secondary antibody for 50 min at room temperature and then washed in three changes of PBS. Mouse immunoglobulin G1 (IgG1) was used as an isotype control antibody in the same diluent as primary antibody for negative control experiments (Supplementary Fig. S12). Dentin matrix protein-1 (DMP-1) was used for positive control experiments. A 10 μg mL^−1^ solution of monoclonal mouse anti-DMP-1 raised against its C-terminal portion (Millipore) and a secondary goat anti-mouse antibody conjugated to Alexa-488 was applied at 2 μg mL^−1^ (Supplementary Fig. S13). Samples were subsequently mounted with Prolong Gold (Invitrogen) and analysed using a Zeiss LSM 710 confocal microscope at sequential 1 μm optical slice intervals.

## Additional Information

**How to cite this article**: Austin, C. *et al.* Uncovering system-specific stress signatures in primate teeth with multimodal imaging. *Sci. Rep.* **6**, 18802; doi: 10.1038/srep18802 (2016).

## Supplementary Material

Supplementary Information
