# Supplementary material for: Uncovering system-specific stress signatures in primate teeth with multimodal imaging
Source: Sci Rep. 2016 Jan 4;6:18802. doi: 10.1038/srep18802 (PMC4698674; doi:10.1038/srep18802)
Supplement: Supplementary Information [file srep18802-s1.pdf]

Uncovering system-specific stress signatures in primate teeth with multimodal imaging

Supplementary Information

Christine Austin[1,2†], Tanya M. Smith[3†], Ramin M. Z. Farahani[2†], Katie Hinde[3,4], Elizabeth A. Carter[5,6], Joonsup Lee[5,6], Peter A. Lay[5,6], Brendan J. Kennedy[6], Babak Sarrafpour[1], Rosalind J. Wright[7], Robert O. Wright[1], Manish Arora[1,2*]

[*] Correspondence: manish.arora@mssm.edu (M.A)

[†] These authors contributed equally

The Supplementary Information includes Supplementary Fig. S1-S13, Supplementary Tables S1-S2 and Supplementary Discussion that provide greater detail on sample preparation and an expanded discussion of the Raman spectroscopy and heat shock protein results.

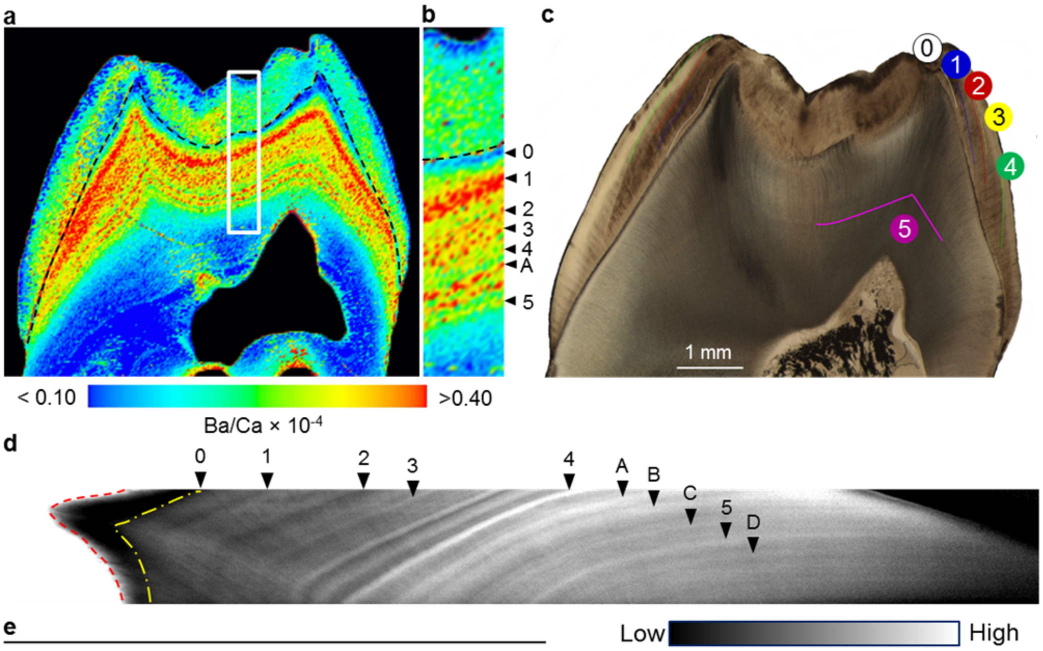

e

Medical history	Timing of stress bands
**(1)** D33: Relocation (dam present)	35-47
**(2)** D93-94: Biobehavioural assessment	82-93
**(3)** No medical note	106
**(4)** No medical note	132-140
**(A)** D166-173: Hospitalisation	
D201: Dam conceived subsequent offspring	
**(5)** No medical note, mother pregnant	224-253
D465-502: Hospitalisation	
D536-561: Hospitalisation, medical euthanasia	

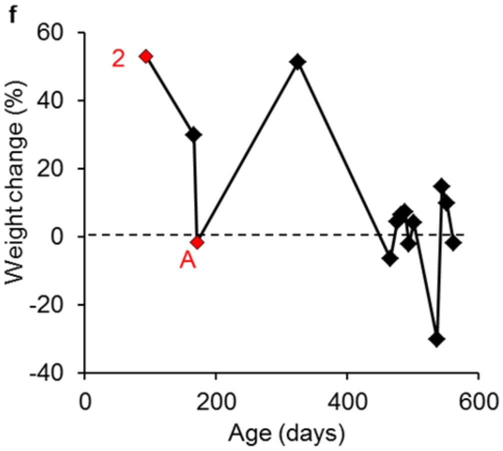

Supplementary Figure S1: Biochemical signatures of MMU515 molar during multiple stress events. (**a**) Ba/Ca distribution map shows multiple bands of increased concentration. Timing of Ba bands was determined by histological analysis. (**b**) Area highlighted in **a**. Discrete bands of high Ba are shown by arrowheads corresponding to this animal's medical history (**e**, **f**). (**c**) Histological light microscopy image. Accentuated lines have been temporally labelled corresponding to medical data in **e**. (**d**) Raman spectroscopy map generated using principle component analysis. Raman analysis was performed on the facing tooth block of the thin section analysed in **a**-**c**. (**e**) Summary of macaque's medical history. Event numbers correspond to data in **b**-**d**. (**f**). Percent weight change over consecutive measurements as proxy for skeletal growth trajectory. Severe disruptions in normal weight gain trajectory are indicated by numbers and are coincident with medical events (**e**) and bands of increased Ba concentration (**b**). The DEJ is indicated by a dashed line.

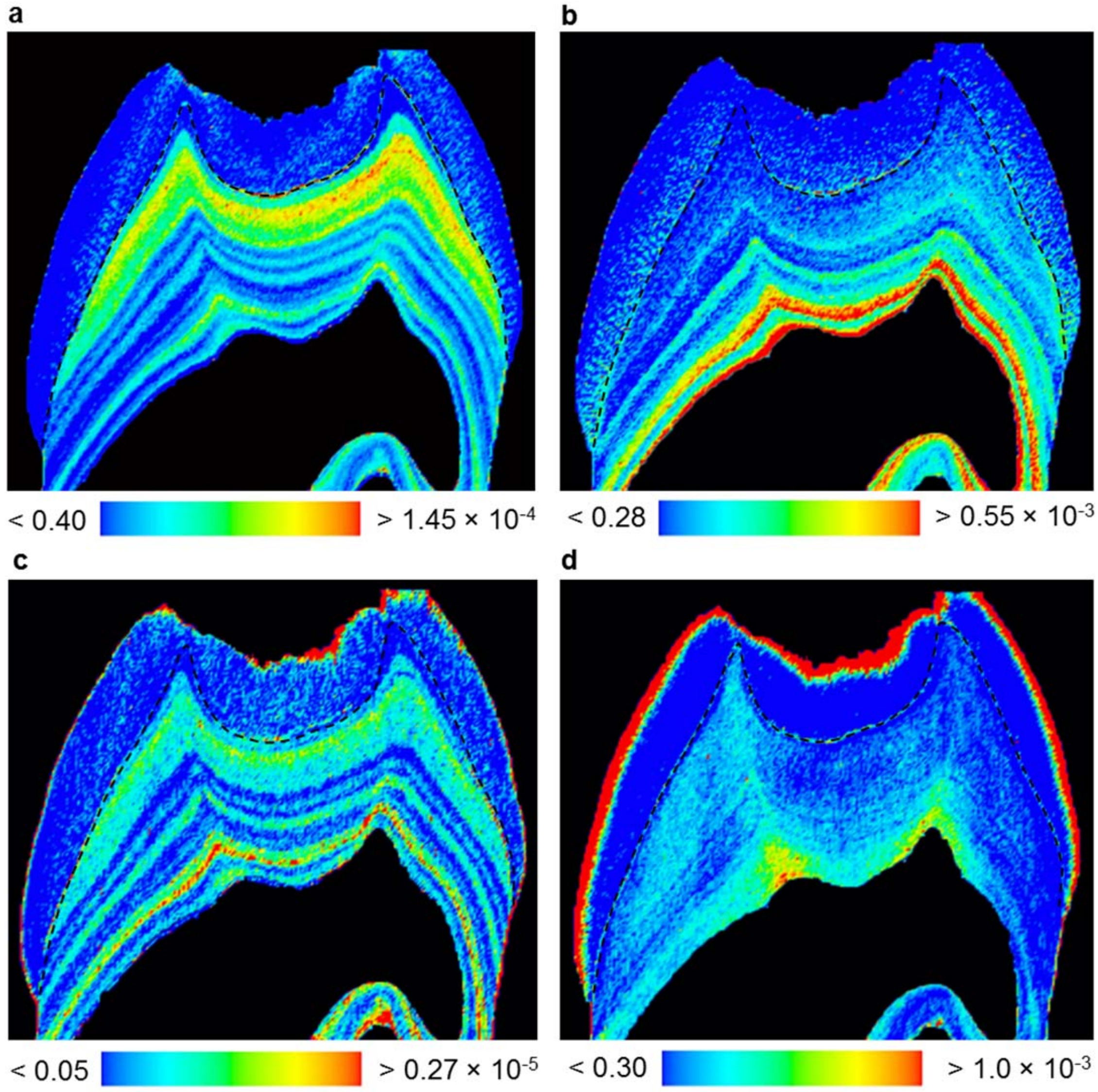

Supplementary Figure S2: Elemental images of MMU401 with the dentine-enamel junction (DEJ) indicated by a dashed black line; (**a**) Ba/Ca, (**b**) Sr/Ca, (**c**) Pb/Ca and (**d**) Zn/Ca.

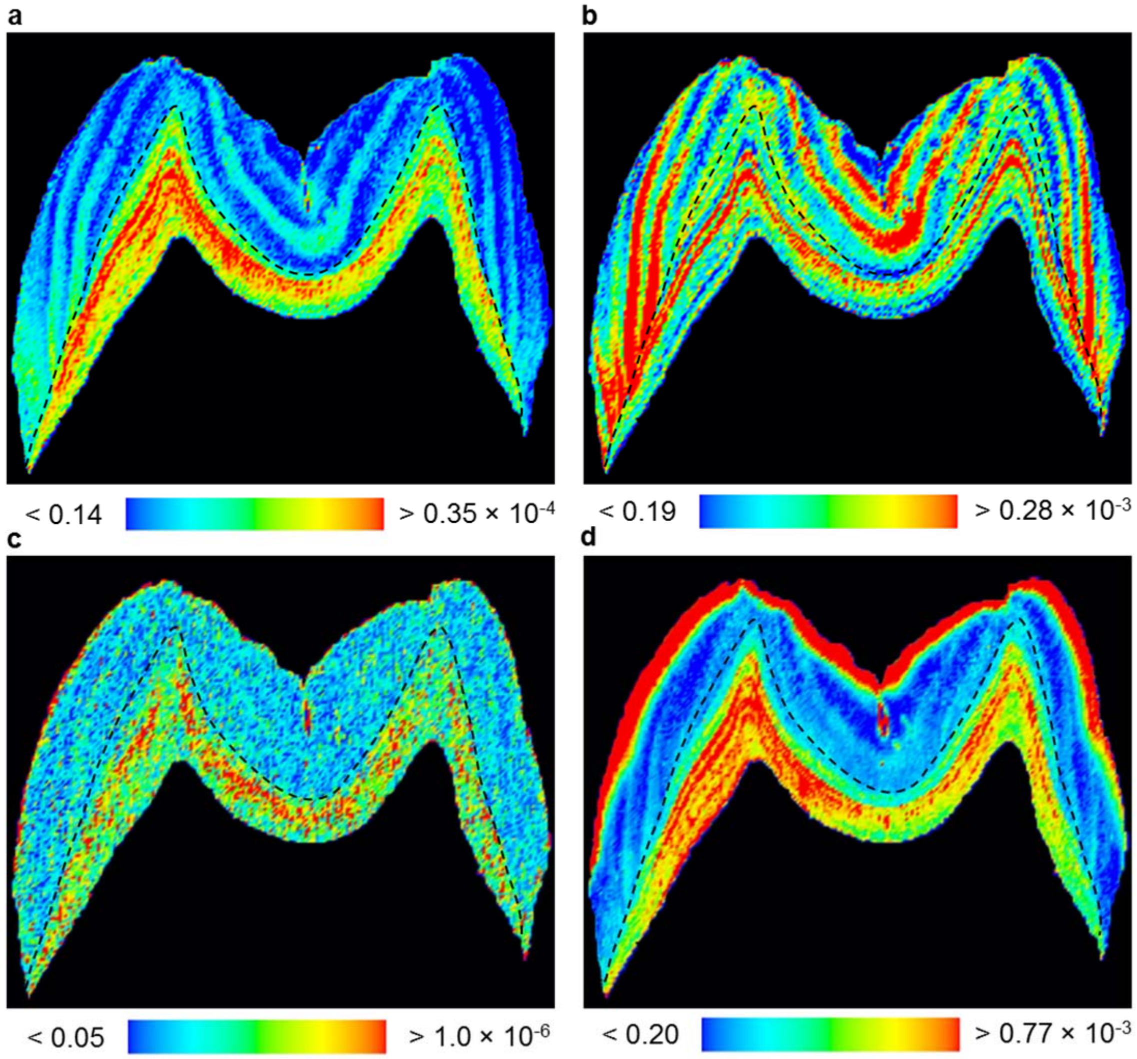

Supplementary Figure S3: Elemental images of MMU336 with the dentine-enamel junction (DEJ) indicated by a dashed black line; (**a**) Ba/Ca, (**b**) Sr/Ca, (**c**) Pb/Ca and (**d**) Zn/Ca.

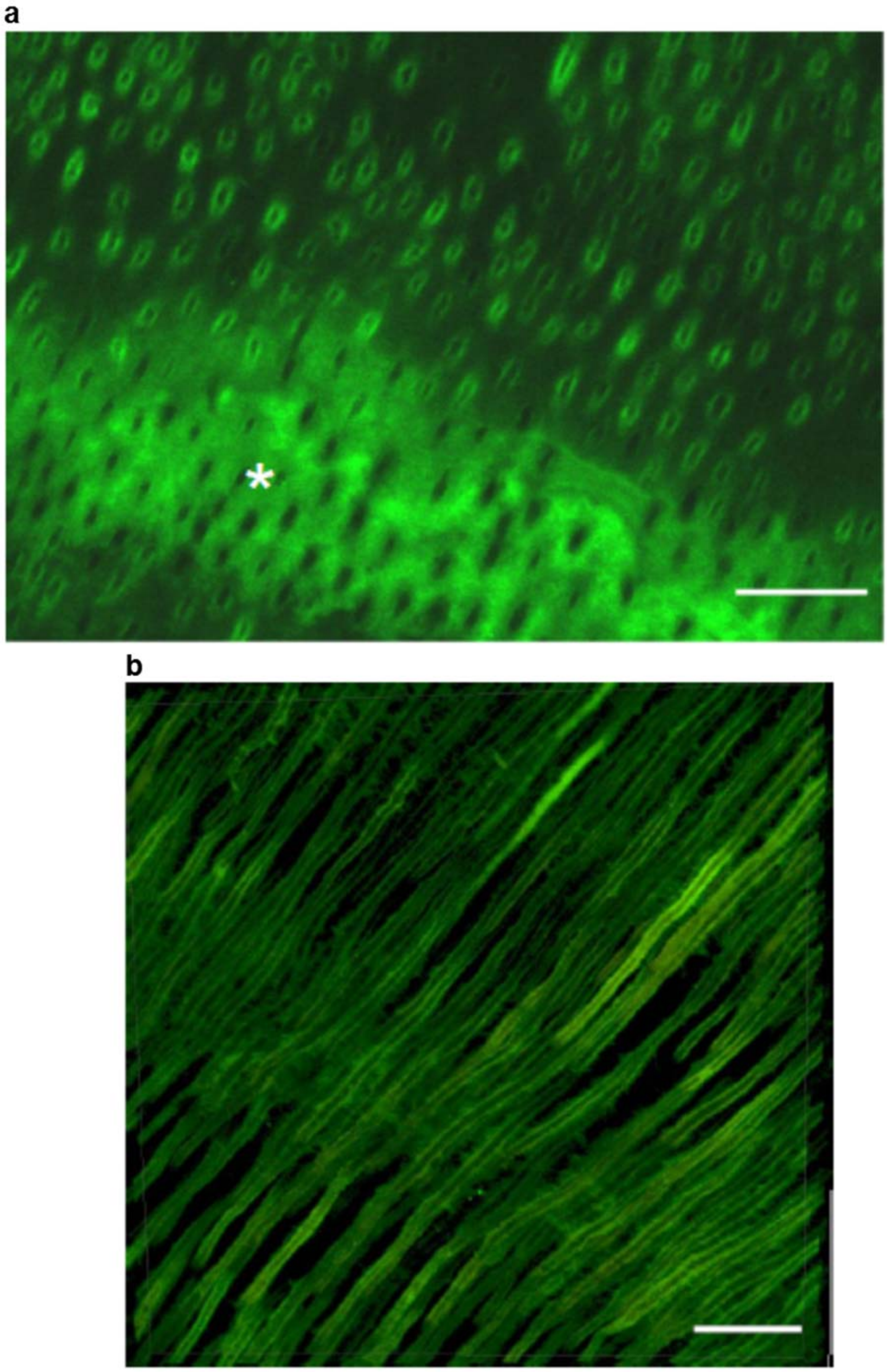

Supplementary Figure S4: Heat shock protein-70 distribution in dentine. (**a**) Cross-sectional view of dentinal tubules shows that HSP70 is deposited on the periphery of dentinal tubules, presumably on the intratubular dentine. Stress-related band of increased HSP70 expression is also seen (*). Image adjustments: brightness +20 %, contrast +20 %. (**b**) Longitudinal view of dentinal tubules showing HSP70 expression is maximal on the wall of the tubules. Scale bar = 20 μm.

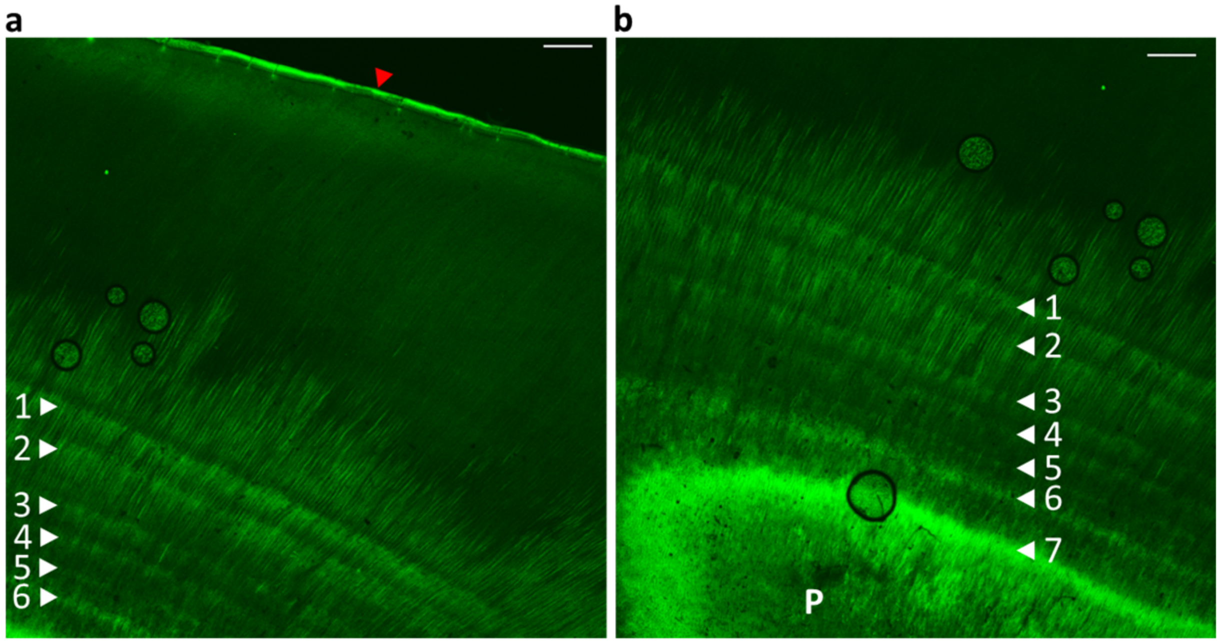

Supplementary Figure S5: Imprint of stress events in dentine identified by heat shock protein-70 expression in MMU401 molar shown in Fig. 1. (**a**) View of fluorescent banding in relation to DEJ, indicated by red arrow head. (**b**) View of fluorescent banding in relation to pulp (P). Banding is labelled relevant to events in Fig. 1. Scale bar = 100 µm. Image adjustments: brightness +20 %, contrast +20 %.

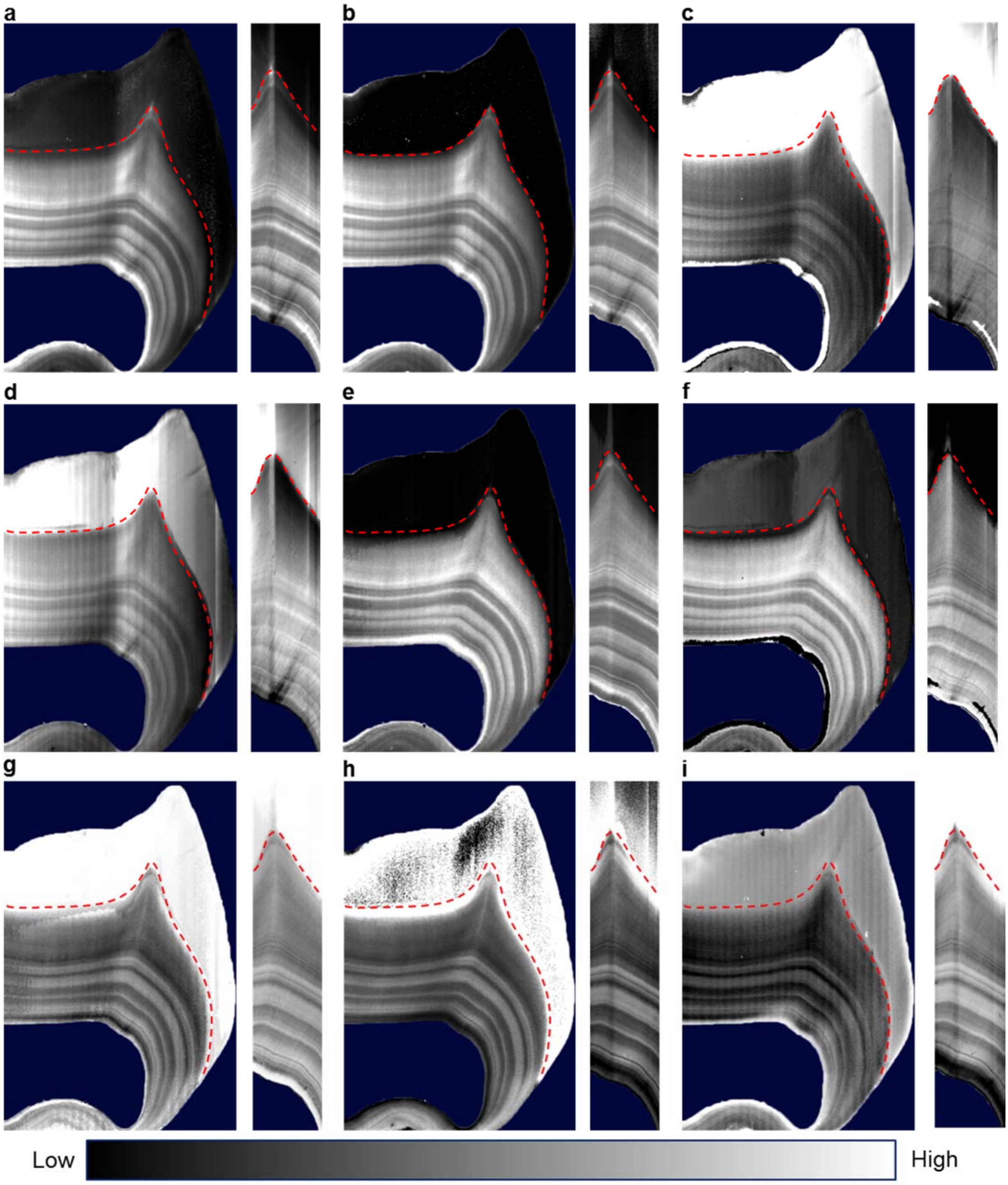

Supplementary Figure S6: Raman spectroscopic maps of MMU401 molar generated using different band parameters; (**a**) 874 cm^{-1} band intensity, (**b**) 1242 cm^{-1} band intensity, (**c**) 1045 cm^{-1} band area (**d**) 1070 cm^{-1} band intensity, (**e**) 959 cm^{-1} band width, (**f**) 1046 cm^{-1} band position, (**g**) band area ratio 959/1070 cm^{-1}, (**h**) band area ratio 959/1242 cm^{-1}, (**i**) principle component analysis (PC 3 left and PC 2 right).The DEJ is indicated by a dashed red line.

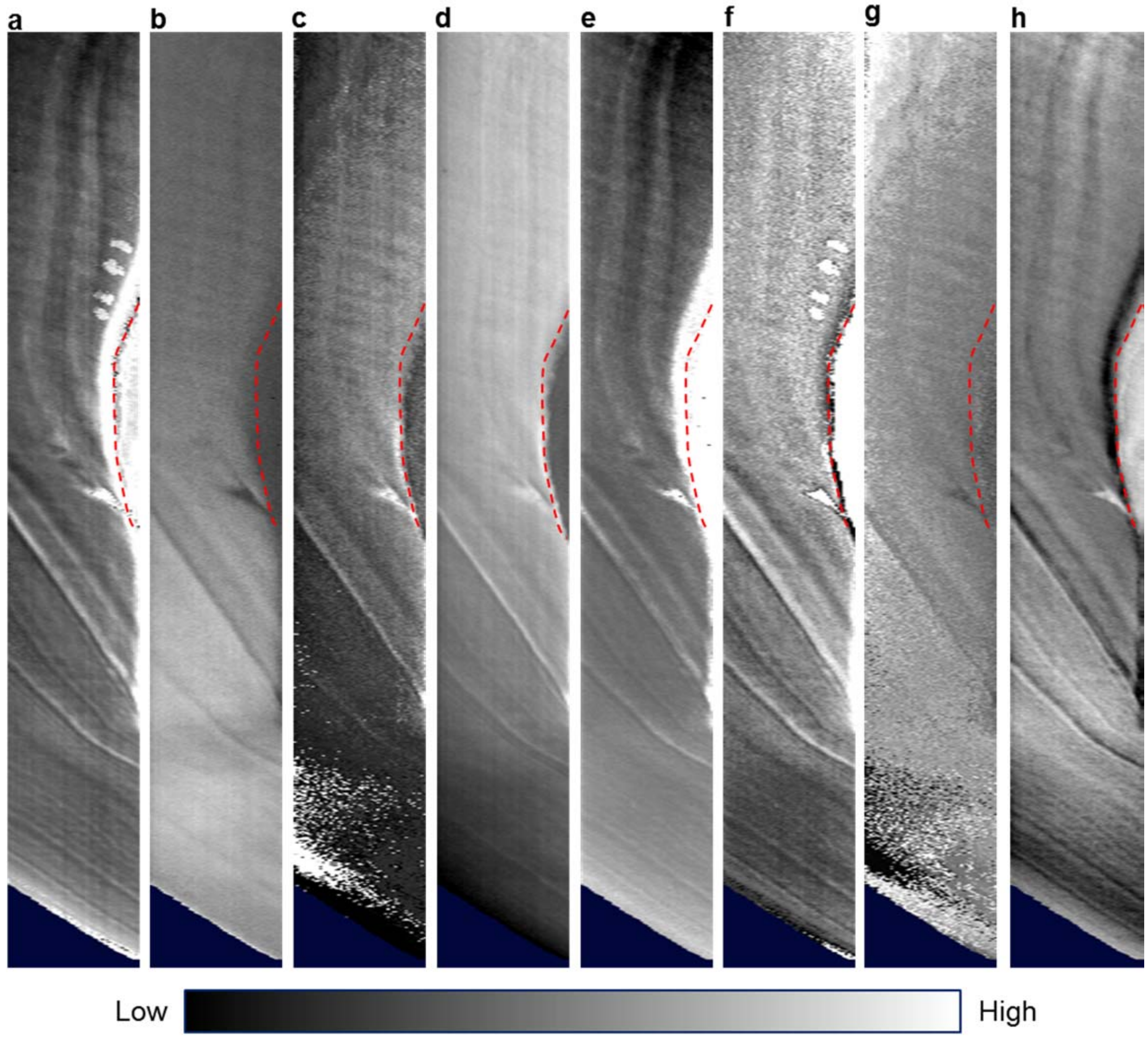

Supplementary Figure S7: Raman spectroscopic maps of MMU336 molar using different band parameters; (**a**) 1388 cm^{-1} band area, (**b**) 812 cm^{-1} band area, (**c**) 1070 cm^{-1} band area, (**d**) 959 cm^{-1} band area, (**e**) 812 cm^{-1} band position, (**f**) 1342 cm^{-1} band width, (**g**) band area ratio 959/1070 cm^{-1}, and (**h**) PC 5 obtained using principle component analysis. DEJ indicated by dashed red line.

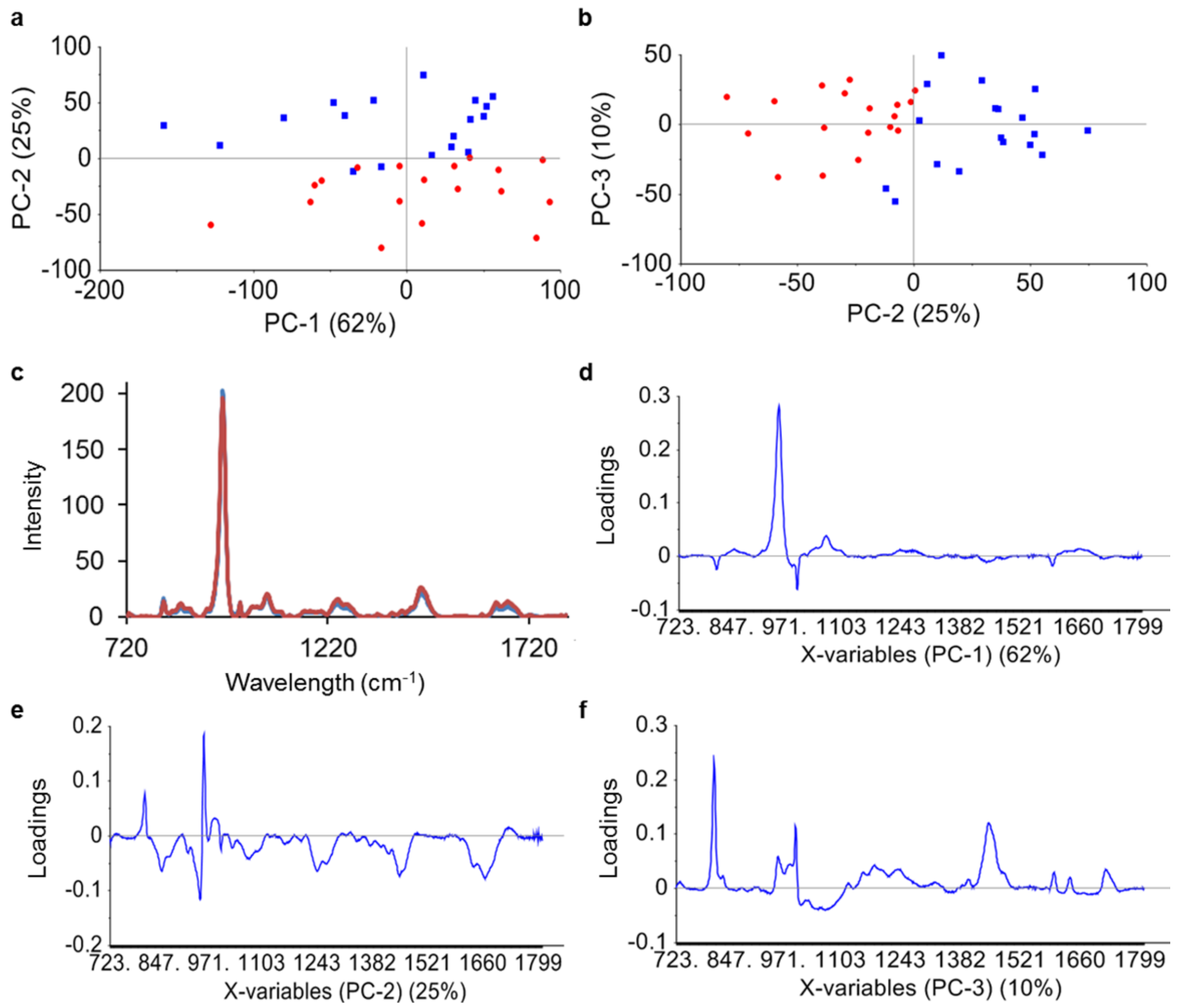

Supplementary Figure S8: Principle component analysis of spectra extracted from a Raman spectroscopy map collected from MMU401. (**a**) PCA scores plotted as PC 1 vs PC 2 and (**b**) PC 2 vs PC 3. (**c**) Averaged spectra extracted from dentine along accentuated lines (red) and adjacent dentine (blue). Loadings plots from (**d**) PC 1, (**e**) PC 2 and (**f**) PC 3.

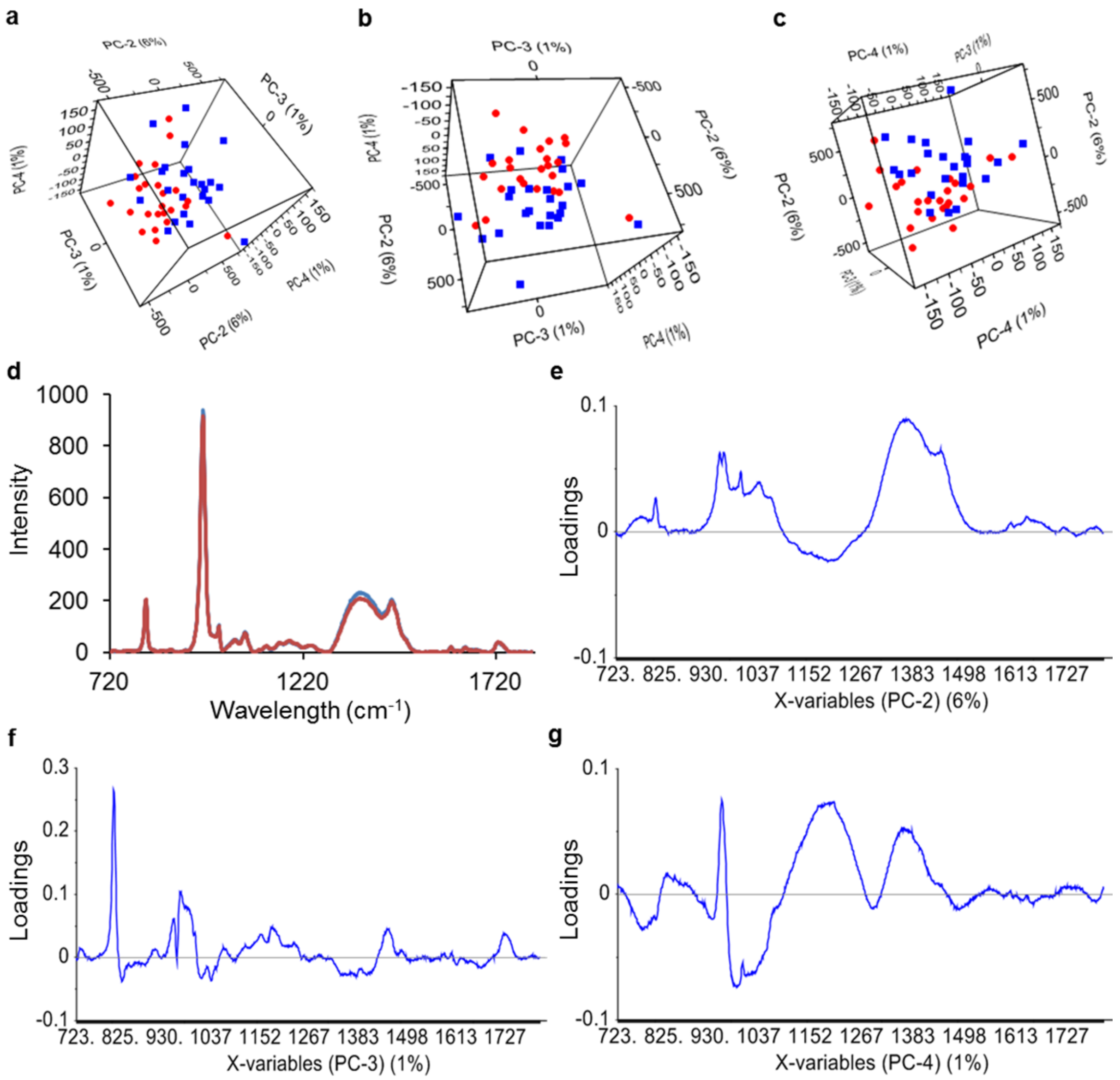

Supplementary Figure S9: Principle component analysis of spectra extracted from a Raman spectroscopy map collected from MMU336. (**a-c**) 3D PCA scores plots of PC 2 vs PC 3 vs PC 4. (**d**) Averaged spectra extracted from dentine along accentuated lines (red) and adjacent dentine (blue). (**e-g**) Loadings plots for (**e**) PC 2, (**f**) PC 3 and (**g**) PC 4.

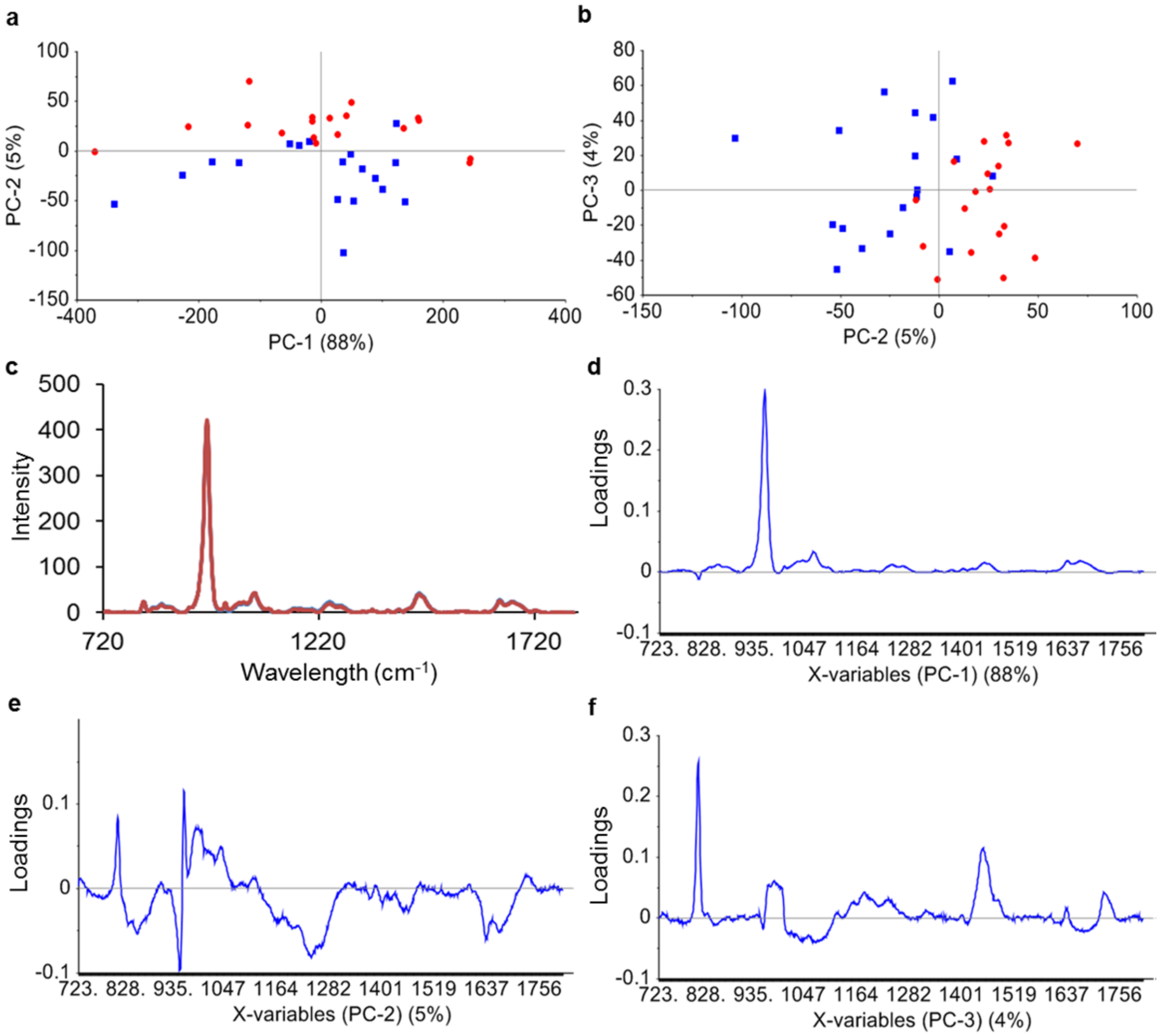

Supplementary Figure S10: Principle component analysis of spectra extracted from a Raman spectroscopy map collected from MMU515. PCA scores plotted as (**a**) PC 1 vs PC 2 and (**b**) PC 2 vs PC 3. (**c**) Averaged spectra extracted from dentine along accentuated lines (red) and adjacent dentine (blue). Loadings plots from (**d**) PC 1, (**e**) PC 2 and (**f**) PC 3.

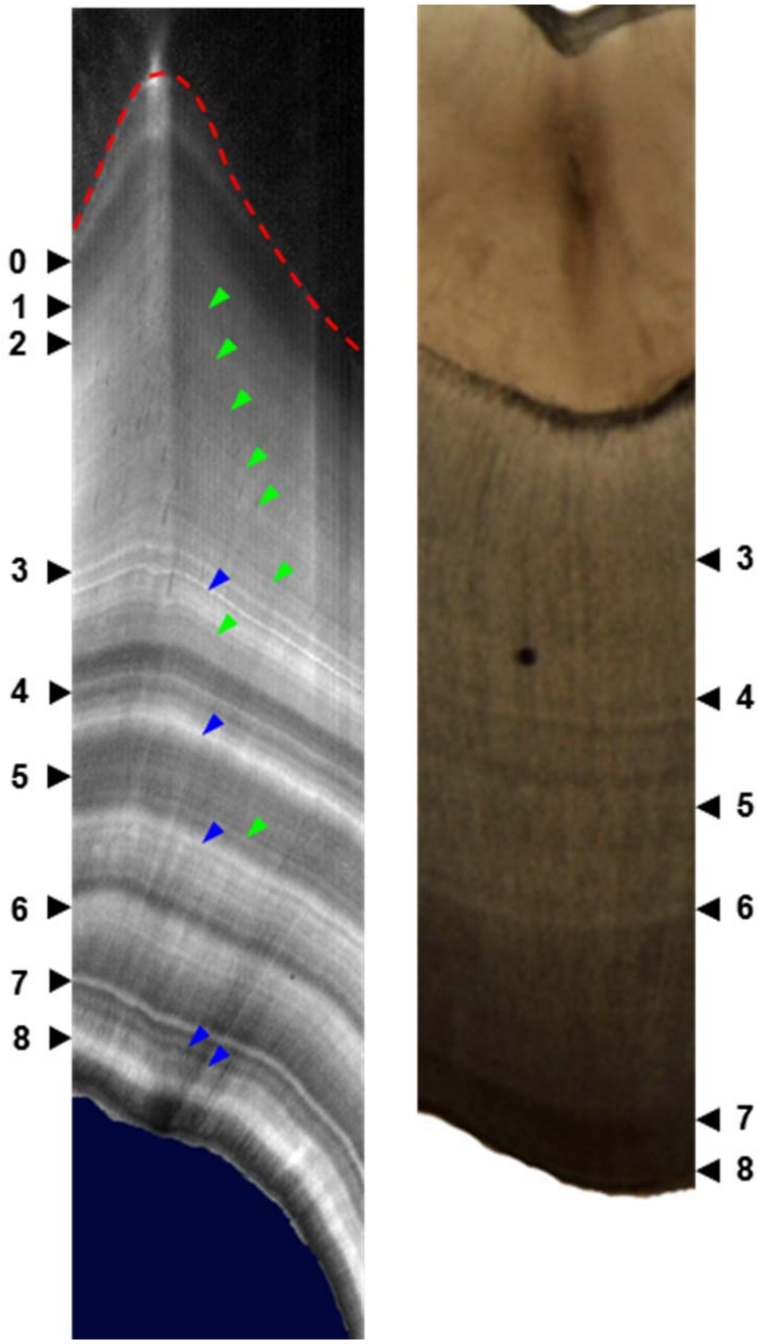

Supplementary Figure S11: Comparison of detail available from MMU401 molar using high resolution Raman spectroscopy (left) and traditional light microscopy method (right). Green arrows indicate regular incremental lines and blue arrows indicate accentuated lines in dentine not observed under light microscopy. The Raman spectroscopy image was taken from the facing tooth block of the thin section analysed in the light microscopy image.

a b

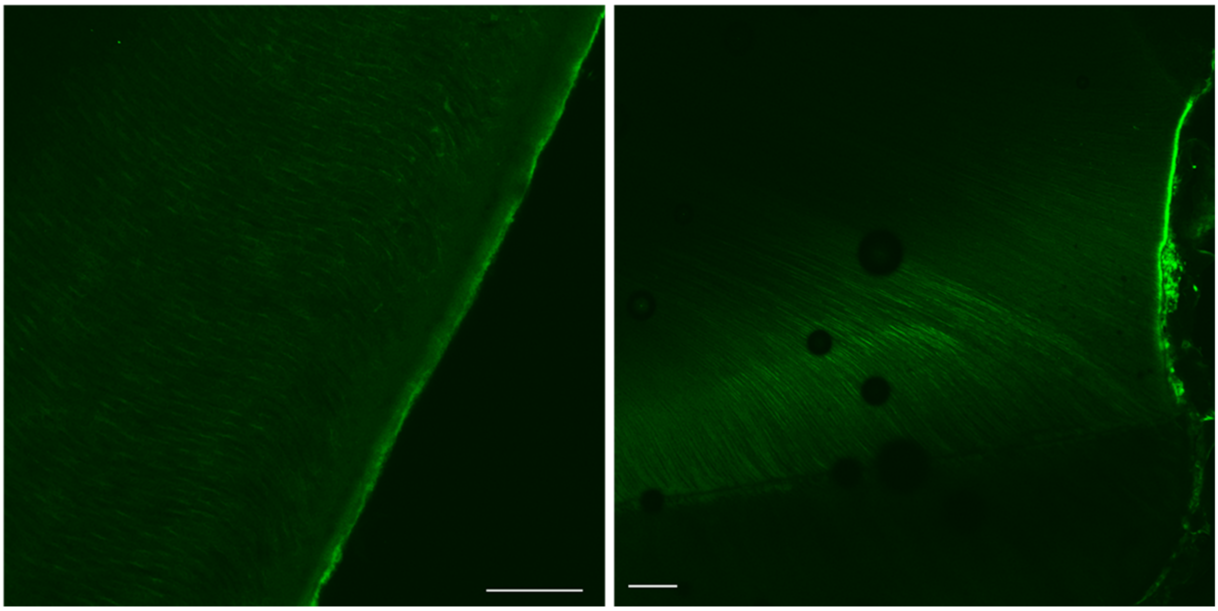

Supplementary Figure S12: Negative control for fluorescent microscopy used in HSP70 experiments. (**a**) Overview of human tooth sample under x20 objective (scale bar is 100 µm) showing auto-fluorescence along the DEJ. (**b**) Overview of human tooth sample under x10 objective (scale bar is 100 µm) showing auto-fluorescence along dentine exposed by attrition at the tooth crown. Dark circles are bubbles under the coverslip. Image adjustments: brightness +20 %, contrast +20 %.

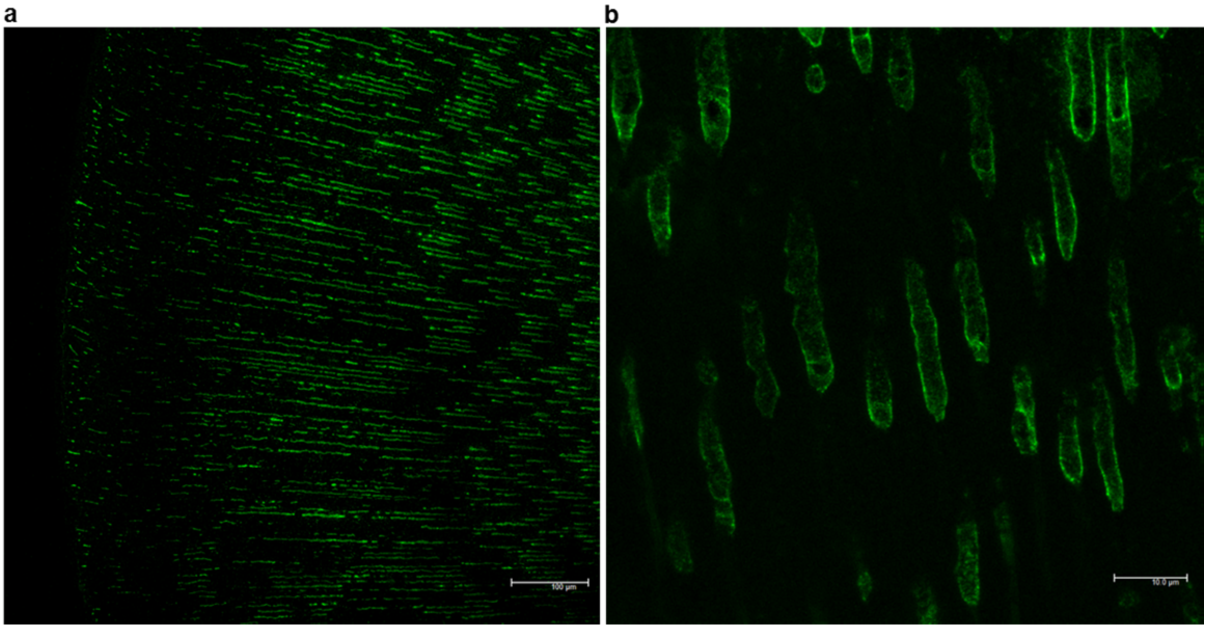

Supplementary Figure S13: Positive control immunohistochemical stain for dentin matrix protein-1 (DMP-1). (**a**) Overview of human tooth sample under x20 objective (scale bar is 100 µm) and (**b**) higher magnification showing distribution of DMP-1 along tubule walls (scale bar is 10 µm). Image adjustments: brightness +20 %, contrast +20 %.

Supplementary Table S1: Comparison of medical records of animals included in this study with accentuated line (AL) age determined by light microscopy and correlation of elemental and Raman accentuated lines

ID	AL age (days)[a]	AL label	Medical records	Elemental AL[b, c]	Raman AL[c, d]
MMU401	0	0	0: Birth	0	0
			12: First physical exam/tattoo		
	25	1	19-33: New social group formation (dam present)	1	1
	51-55	2		2	2
	142-151	3		3	3
	163<br>179-189<br>199	4	166-194: Hospitalization	4	4
	223-231	5	217-232: Hospitalization	5	5
			267-292: Hospitalization		H
	293-301	6	306-313: Hospitalization	6	6
	363	7	357-376: Hospitalization	7	7
	403	8	402: Medical euthanasia	8	8
MMU336	1116				
	1133				
	1146		1147-1187: Hospitalization		
	1153-1166	1		1, 2	1
		2			2
	1210	3		3, 4	3
	1220-1231	4	1222-1277: Hospitalization		4
	1275-1291	5	1273-1291: Hospitalization	5	5
	1301-1317		1317: Conception 1		C
	1319-1327	6		6, 7	6
	1335	7			7
	1365-1373	8	1364-1382: Hospitalization<br>1367: Spontaneous abortion	8	8
	1387-1394	9		9	9
			1410-1413: Hospitalization		
			1413: Medical euthanasia		
MMU515	0		0: Birth	0	0
			10: First physical exam/tattoo		
	35-47	1	33: 8-hr Relocation (dam present)	1	1
	82-93	2	93-94: 25-hr Biobehavioural assessment	2	2
	106	3		3	3
	132-140	4		4	4
			166: Hospitalization (dam present)	A	A
			179: Relocation (dam present)		B
			192: New social group formation (dam present)		

			201: Dam conceived subsequent offspring		
	224-253	5		5	5
			465-502: Hospitalization		
			536: Hospitalization		
			561: Medical euthanasia		
MMU152	0		0: Birth	0	
	46	1			1
			79-80: 25-hr Relocation (Biobehavioural assessment control subject)		
	91	2			2
	105	3			3
	114				
	133-135				
	167				
	185-193	4		4	4
	207-209	5		5	5
	255-261	6	256: First physical exam/tattoo<br>257-282: Hospitalization	6	6
			469: Hospitalization		H
			487: Medical euthanasia		
MMU325	0	0	0: Birth, first physical exam/tattoo	0	0
	35	1		1	
	54	2		2	
	74-90[e]	3	90-91: 25-hr Biobehavioural Assessment	3	
	127	4	124-137: Hospitalization	4	4
	164	5	165-179: Hospitalization	5	5
	202	6	190-207: Hospitalization	6	6
	230		220: Hospitalization	H	H
	250		221: Medical euthanasia		
MMU151	0	0	0: Birth	0	
	104[e]	1	92: First physical exam/tattoo<br>105-106: 25-hr Biobehavioural Assessment	1	
	134	2	139-168: Hospitalization (dam present)	2	2
	169		171: Dam conceives subsequent offspring		
	192	3		3	3
	206		206: Tattoo redone		
	234	4		4	4
	258		271-288: Hospitalization		
	294	5	288: Housed indoors	5	5
	300				
	308				

	350	6			
	382	7			
	442<br>454	8		8	
			469: Relocation		
	482		477: Medical euthanasia		
MMU619	0	0	0: Birth		NA
	9-12		14-19: Hospitalisation		
			32: 8-hr Relocation (dam present)		
	46-52				
	68-75				
	108-114	1	95-96: 25-hr Biobehavioural assessment		
			124: Physical exam, tattoo		
	138				
	162		154: 8-hr Dam relocated		
	188-191	2		2	
	201				
	221-225		217: Dam conceives subsequent offspring		
	249-258				
	271-286	3	285-301: Hospitalisation	3	
	299-302<br>299-308	4		4	
			424: New social group formation (dam absent)		
			481: Hospitalisation		
			540: Medical euthanasia		
MMU473	0	0	0: Birth	0	NA
	36-46				
	55-77		60: First physical exam/tattoo		
	103-111		104-105: 25-hr Biobehavioural assessment		
	111-130				
	153				
	169-209		186: Relocation, indoor<br>187: Euthanasia (research)		
MMU542	0	0	0: Birth	0	NA
	52-74	1	70: First physical exam/tattoo	1	
	93-116		92-93: 25-hr Biobehavioural assessment		
	121-169	2		2	
	185-261	3	186: Hospitalisation, medical euthanasia	3	

[a] Ranges indicate multiple estimates of the same event in different cusps.
[b] Identification of elemental AL was performed by overlaying elemental and aged light microscopy maps.

[c] Uppercase letters refer to AL observed in the elemental and Raman maps that were not observed or aged in the light microscopy map.
[d] Identification of Raman AL was performed by overlaying elemental and Raman maps or Raman and light microscopy maps of the same section. Because many of the Raman maps were taken from the facing tooth section, the aging of Raman AL is less precise.
[e] There is some uncertainty regarding timing of these AL across different cusps and therefore correspondence with the event is only potential

Supplementary Table S2: Raman spectroscopy band assignments[59-61].

**Assignment**	**Association**	**Raman Shift (±1 cm^{-1})**
ν(CC), ν(COC)	Collagen	812
δ(CCH) aromatic, proline	Collagen	854
ν(CC), hydroxyproline	Collagen	874
$\nu_1(PO_4^{3-})$	Phosphate	959
$\nu_2(PO_4^{3-})$	Phosphate (b-type carbonate substitution)	1031
$\nu_3(PO_4^{3-})$ out of phase, $\nu_3(CO_3^{2-})$	Phosphate (b-type carbonate substitution), carbonate	1045
$\nu_1(CO_3^{2-})$	Carbonate (b-type)	1070
δ(NH)	Amide III	1242
δ(NH)	Amide III	1270
δ(CH)	Amide I	1424
δ(CH) deformation	Amide I	1449
δ(CH)	Amide I	1463
ν(CC), ν(C=O)	Amide I (β sheets)	1637
ν(C=O)	Amide I (α-helix)	1666
ν(C=O)	Amide I (turns)	1687

ν – stretching, δ – bending,

Supplementary discussion

Sample Selection

Thin sections were required to age accentuated lines in enamel and dentine by light microscopy. Elemental maps using laser ablation-inductively coupled plasma-mass spectrometry (LA-ICP-MS), were collected on the same sections. However, Raman spectroscopy could not be directly performed on sections from MMU401, MMU515, MMU152 and MMU619 due to fluorescence from the underlying glass slide interfering with the sample signal. We performed Raman spectroscopy analysis on the facing tooth block of these thin sections. We used the dentine-enamel junction and neonatal line to estimate timing of accentuated lines observed in Raman spectroscopy maps and compared these to accentuated lines temporally labelled in the corresponding light microscope images and elemental maps.

Expanded Raman Spectroscopy Discussion

Raman spectroscopy has been used to examine the molecular structure of different tissues within teeth (e.g. peritubular/intratubular dentine[60], dentine-enamel junction[62]), and changes induced by external agents (laser irradiation[63], physical indentations[64], external ionizing radiation[65]) and oral diseases (fluorosis[66], devitalization of teeth[67], non-carious cervical lesions[68] and caries[69,70]). Bands in the Raman spectrum are assigned to specific molecular structures (Supplementary Table S2) based on the available literature. Changes in the band area or intensity are attributed to changes in the molecular environment, i.e. the other chemical bonds in proximity. The Raman spectrometer used in this study has previously been reported to have an accuracy of ± 0.05 cm^{-1} and therefore very small changes in band shift can be accurately measured[64]. By mapping band area, intensity, width and position, we can explore the changes in

the molecular structure across the mapped area and in this case, observe accentuated lines due to small changes in these band parameters.

Pre-processing tools such as baseline correction and normalization are typically applied to Raman spectra to remove variance in the spectra. However, there are currently no standard procedures recommended. We generated Raman maps with and without polynomial baseline correction of the raw data, followed by calculation of the second derivative to remove any sloping baseline and aid in resolving spectral windows that contained numerous overlapping bands. Comparison of these two different data sets revealed no marked difference in the quality of images in regards to the clarity of accentuated lines.

Accentuated lines were clearly visible in maps generated by calculating the area, intensity, width or position of different bands. This indicates differences exist in the molecular structure of dentine/enamel deposited along accentuated lines and adjacent tissue. The clarity of accentuated lines in Raman spectroscopy maps varied depending on the band parameter selected to generate the map. In addition, maps that were generated with a particular band parameter that produced a map showing accentuated lines were not found to produce consistent results across all samples and did not show the same trends in all samples. For example, accentuated lines in MMU401 were visible as an increase in the intensity of the amide III band at 1242 cm^{-1} but a decrease in the same band intensity of spectra collected from sample MMU515, and accentuated lines were visible as both increases and decreases in the phosphate/carbonate band area ratio in MMU401 maps (see Supplementary Fig. S6).

We used principle component analysis (PCA) to examine spectra extracted from dentine/enamel deposited along accentuated lines to establish if the data could be objectively

classified as a separate group from adjacent tissue. Loadings plots were also expected to give an indication of where the differences in spectra occurred.

Gaigneaux *et al.*[71] studied the effect of different pre-processing methods on PCA of Raman spectra and found that baseline correction and normalization improved spectrum classification but use of first or second derivatives was not recommended for PCA. We ran PCA on spectra extracted from accentuated lines and adjacent tissue of MMU401, MMU336 and MMU515 samples, with and without baseline correction. Either method showed grouping of dentine/enamel deposited along accentuated lines and adjacent tissue, however greater overlap was observed for MMU336 enamel.

The Raman analysis of enamel in samples MMU336, MMU325 and MMU151 was complicated by fluorescence of the glass substrate the samples were mounted on and the change in enamel density across the immature enamel. The enamel spectrum from these samples was very different with an intense, broad band observed in the 1500 – 1300 cm^{-1} spectral range which was not observed in the Raman spectra from other samples. The underlying adhesive used to fix the sample to the slide is a major contributor to this band. However, accentuated lines are clearly observed in maps of this band range. Further investigation is required to separate the signal from the accentuated lines and that from an interaction of the sample with the adhesive.

Expanded Heat Shock Proteins Discussion

Heat shock proteins are evolutionary highly conserved molecular chaperones. For example, the major HSP70 from Escherichia coli, DnaK, shares around 50 % amino acid identity with eukaryotic HSP70 proteins[37,72]. While some heat shock proteins, such as HSP27 and HSP70 are inducible, released at higher levels in response to environmental and physiological stressors, others, such as HSP90, are constitutively expressed[38,39].

Glucose related protein 78 kDa (GRP78 or HSPA5) is a member of the HSP70 family. GRP78 is a membrane receptor for endocytosis dentin matrix protein-1 (DMP-1)[73,74], an essential regulator of dentine mineralization[75]. DMP-1 is present in odontoblasts, dentinal tubules and ameloblasts, and regulates DSPP gene transcription[76]. DMP-1 is also involved in the biomineralization of bones[77], phosphate homeostasis[78], and differentiation of odontoblasts and osteoblasts[79]. DMP-1 null mice show a distinct phenotype characterized by maturation defects of dentin and hypomineralization[80].

Considering the evolutionary conservation and similar properties of GRP78 and other HSP70 isoforms, stress-induced HSP70 may act as a decoy receptor for DMP-1 and hence alter the structure of dentin by sequestering the odontoblastic DMP-1. We speculated that the proposed bimodal switch may act as a biological marker of early life stressors with signatures embedded into the dentin matrix.

HSP-70 is a molecular trap with open and closed conformations. Binding affinity of HSP70 to other proteins is inversely proportional to [ATP]/[ADP][81]. When excess ADP is present, mammalian HSP70 binds tightly to other proteins. Upon restoration of ATP concentration, structure of HSP70 changes from close to open conformation following hydrolysis of ATP[81]. Hence considering the structural and functional similarity of HSP70 to GRP78 (endocytotic receptor of DMP-1), one may argue that stress-induced HSP70 release by odontoblasts may mimic GRP-78 and hence sequester DMP-1. Microanatomical similarity of dentin tubular disruption to dentinal features of *Dmp-1* null mice[80] bolsters this hypothesis.

Image Processing

Background in light micrographs in Fig. 1 and 2 (panel C) was deleted for clarity and to allow labelling. Backgrounds of elemental maps were converted to black (absent from the color

intensity scale) to clarify sample boundaries from the substrate. Elemental maps were rotated and black boarders added where needed to align rectangular figures. Backgrounds of Raman spectroscopy maps were converted to blue (absent from the grey scale) to clarify sample boundaries from the substrate. The width of the Raman spectroscopy map in Fig. 2D was increased to better enable visualization of Raman accentuated lines within the confines of the figure panel. Image contrast and brightness was adjusted in Supplementary Fig. S3A, as noted in the corresponding figure legend. Brightness of the light micrograph in Fig. 2C was also adjusted (+50).

Additional References

59. H.-U. Gremlich, B. Yan, *Infrared and Raman Spectroscopy of Biological Materials* (Marcel Dekker, New York, 2001).

60. C. Xu, Y. Wang, Chemical composition and structure of peritubular and intertubular human dentine revisited. *Arch. Oral Biol.* **57**, 383 (2012).

61. M. T. Kirchner, H. G. M. Edwards, D. Lucy, A. M. Pollard, Ancient and modern specimens of human teeth: A Fourier transform Raman spectroscopic study. *J. Raman Spectrosc.* **28**, 171 (1997).

62. E. Wentrup-Byrne, C. A. Armstrong, R. S. Armstrong, B. M. Collins, Fourier transform Raman microscopic mapping of the molecular components in a human tooth. *J. Raman Spectrosc.* **28**, 151 (1997).

63. G. Jegova, R. Titorenkova, M. Rashkova, B. Mihailova, Raman and IR reflection micro-spectroscopic study of Er:YAG laser treated permanent and deciduous human teeth. *J. Raman Spectrosc.* **44**, 1483 (2013).

64. L.-H. He, E. Carter, M. Swain, Characterization of nanoindentation-induced residual stresses in human enamel by Raman microspectroscopy. *Analytical and Bioanalytical Chemistry* **389**, 1185 (2007).

65. L. A. Darchuk, L. V. Zaverbna, A. Worobiec, R. Van Grieken, Structural features of human tooth tissues affected by high dose of external ionizing radiation after nuclear catastrophe of Chernobyl plant. *Microchemical Journal* **97**, 282 (2011).

66. V. Zavala-Alonso, J. P. Loyola-Rodriguez, H. Terrones, N. Patino-Marin, G. A. Martinez-Castanon, K. Anusavice, Analysis of the molecular structure of human enamel with fluorosis using micro-Raman spectroscopy. *Journal of Oral Science* **54**, 93 (2012).

67. K. Zelic, P. Milovanovic, Z. Rakocevic, S. Askrabic, J. Potocnik, M. Popovic, M. Djuric, Nano-structural and compositional basis of devitalized tooth fragility. *Dent. Mater.* **30**, 476 (2014).

68. K. Karan, X. Yao, C. Xu, Y. Wang, Chemical profile of the dentin substrate in non-carious cervical lesions. *Dent. Mater.* **25**, 1205 (2009).

69. B. Levallois, E. Terrer, Y. Panayotov, H. Salehi, H. Tassery, P. Tramini, F. Cuisinier, Molecular structural analysis of carious lesions using micro-Raman spectroscopy. *Eur. J. Oral Sci.* **120**, 444 (2012).

70. P. Seredin, V. Kashkarov, A. Lukin, Y. Ippolitov, R. Julian, S. Doyle, Local study of fissure caries by Fourier transform infrared microscopy and X-ray diffraction using synchrotron radiation. *J. Synchrotron Radiat.* **20**, 705 (2013).

71. A. Gaigneaux, J. M. Ruysschaert, E. Goormaghtigh, Cell discrimination by attenuated total reflection Fourier transform infrared spectroscopy: the impact of preprocessing of spectra. *Applied Spectroscopy* **60**, 1022 (2006).

72. C. G. Evans, L. Chang, J. E. Gestwicki, Heat shock protein 70 (hsp70) as an emerging drug target. *Journal of medicinal chemistry* **53**, 4585 (2010).

73. S. Ravindran, K. Narayanan, A. S. Eapen, J. Hao, A. Ramachandran, S. Blond, A. George, Endoplasmic reticulum chaperone protein GRP-78 mediates endocytosis of dentin matrix protein 1. *J. Biol. Chem.* **283**, 29658 (2008).

74. C. Qin, R. D'Souza, J. Q. Feng, Dentin matrix protein 1 (DMP1): new and important roles for biomineralization and phosphate homeostasis. *Journal of Dental Research* **86**, 1134 (2007).

75. E. Beniash, A. S. Deshpande, P. A. Fang, N. S. Lieb, X. Zhang, C. S. Sfeir, Possible role of DMP1 in dentin mineralization. *Journal of structural biology* **174**, 100 (2011).

76. K. Narayanan, S. Gajjeraman, A. Ramachandran, J. Hao, A. George, Dentin matrix protein 1 regulates dentin sialophosphoprotein gene transcription during early odontoblast differentiation. *The Journal of biological chemistry* **281**, 19064 (2006).

77. A. George, B. Sabsay, P. A. Simonian, A. Veis, Characterization of a novel dentin matrix acidic phosphoprotein. Implications for induction of biomineralization. *The Journal of biological chemistry* **268**, 12624 (1993).

78. J. Q. Feng, L. M. Ward, S. Liu, Y. Lu, Y. Xie, B. Yuan, X. Yu, F. Rauch, S. I. Davis, S. Zhang, H. Rios, M. K. Drezner, L. D. Quarles, L. F. Bonewald, K. E. White, Loss of DMP1 causes rickets and osteomalacia and identifies a role for osteocytes in mineral metabolism. *Nature genetics* **38**, 1310 (2006).

79. A. Almushayt, K. Narayanan, A. E. Zaki, A. George, Dentin matrix protein 1 induces cytodifferentiation of dental pulp stem cells into odontoblasts. *Gene therapy* **13**, 611 (2006).

80. L. Ye, M. MacDougall, S. Zhang, Y. Xie, J. Zhang, Z. Li, Y. Lu, Y. Mishina, J. Q. Feng, Deletion of dentin matrix protein-1 leads to a partial failure of maturation of predentin into dentin, hypomineralization, and expanded cavities of pulp and root canal during postnatal tooth development. *The Journal of biological chemistry* **279**, 19141 (2004).

81. K. Liberek, D. Skowyra, M. Zylicz, C. Johnson, C. Georgopoulos, The Escherichia coli DnaK chaperone, the 70-kDa heat shock protein eukaryotic equivalent, changes conformation upon ATP hydrolysis, thus triggering its dissociation from a bound target protein. *The Journal of biological chemistry* **266**, 14491 (1991).
